# Characterization of newly identified N–S shear zones in the Egyptian Nubian Shield by integrating geophysical, remote sensing and field data

**DOI:** 10.1038/s41598-026-46327-x

**Published:** 2026-05-25

**Authors:** Mohamed A. Abd El‑Wahed, Ahmed M. Eldosouky

**Affiliations:** 1 Geology Department, Faculty of Science, Tanta University, P.O. Box: 31527, Tanta, Egypt; 2 Department of Geology, Faculty of Science, Suez University, P.O. Box: 43221, Suez, Egypt; 3 Geology of Petroleum and Natural Gas Program, Faculty of Science, Suez National University (SNU), P.O. Box: 43221, Suez, Egypt

**Keywords:** Egyptian Nubian Shield, N-S shear zones, Najd Fault System, Southern Compressional Domain, The Central Transpressional Domain, The Northern Extensional Domain, Keraf Suture, Solid Earth sciences, Geology, Geophysics, Tectonics

## Abstract

This study provides a comprehensive analysis of the large-scale north-south (N-S) shear zones in the Egyptian Nubian Shield (ENS), a critical segment of the East African Orogen. Integrating aeromagnetic, gravity, remote sensing, and field-based structural data, the study delineates the geometry, kinematics, and tectonic significance of these N-S shear zones and their interaction with other major tectonic features, such as the Najd Fault System and the Keraf suture. The ENS is subdivided into compressional, transpressional, and extensional domains, each characterized by distinct structural regimes and evolutionary histories. Within the Central Transpressional Domain, the Najd Fault System (NFS) creates a complex network of NW-SE-trending sinistral strike-slip shear zones intersected by NE-SW-directed dextral shear zones, forming a conjugate shear system. Key large-scale N-S-trending shear zones in the ENS include the Safaga-Shalul, Wadi Kareim-Umm Bisilla, Um Gheig-Nugrus, Barramiya-Mueilha, Abu Swayel-Muqsim, and Himitrah-Madari shear zones. The structural evolution of the ENS comprises five main deformational phases. D1 features N-S shortening, resulting in thrust imbrication and E-W-trending foliations. D2 is marked by NE-SW-directed shortening and NW-trending sinistral shearing, characterized by large-scale sinistral shear zones. The Najd Fault System (620-540 Ma) dominates the Central Transpressional Domain. D3 is characterized by E-W shortening and N-S dextral shearing, leading to the formation of N-S transpressional shear zones and folds (600-590 Ma) that affect all rock units except post-tectonic granites. D4 features N-S-oriented dextral shear zones that affect E-W, NW-SE, and N-S structural fabrics within transpressional belts. Red Sea rifting reactivated these zones, transforming them into brittle, sinistral strike-slip faults that affect all rock units. The NW-SE, N-S, and NE-SW-oriented shear zones in the ENS form a conjugate system linked to the activity of the Najd Fault System. Geophysical data indicate that these shear zones likely extend subsurface, connecting to the extensive N-S-striking shear zones in the Nubian Shield. The Keraf shear zone developed contemporaneously with the NFS, suggesting a link to the genesis of the N-S dextral shear zones.

## Introduction

Large-scale strike-slip faults are essential components of the continental lithosphere. They separate continental plates and terranes with different geological histories within accretionary orogens^1–8^. In some cases, these faults or shear zones connect terranes that exhibit different geological histories. In contrast, extensive strike-slip shear zones typically facilitate relative movement between blocks with comparable geological characteristics^9^. Shear zones are essential for managing tectonic strain, facilitating displacement, and enabling fluid movement within the lithosphere^10–16^. Individual shear zones are typically characterized as flat bands of deformation bounded by less-deformed or undeformed rocks^17^. However, these zones can frequently exhibit nonplanar characteristics and interconnect in complex anastomosed networks, delineating undeformed or less deformed areas^18,19^.

**Fig. 1:**
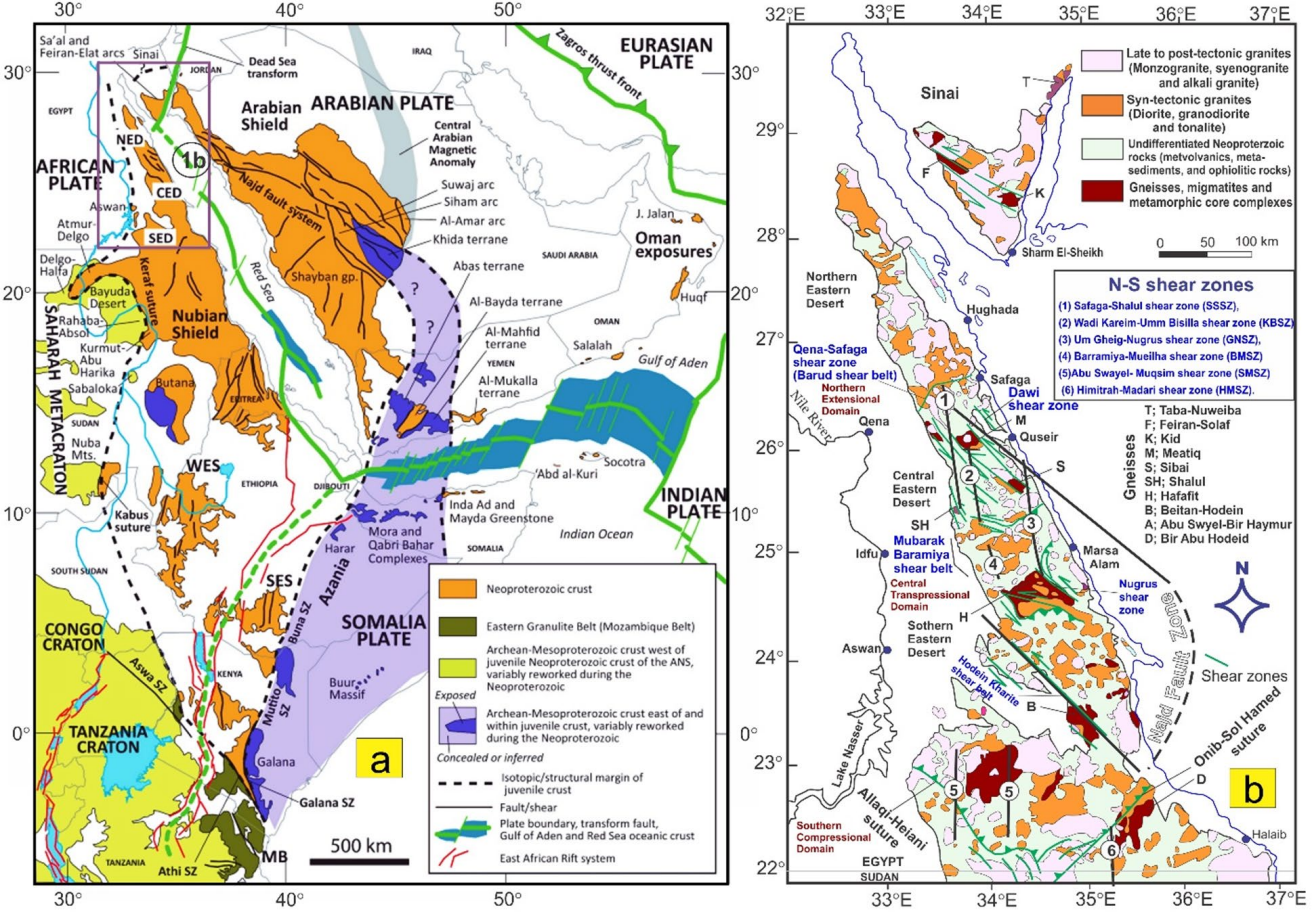
(**a**) Map of the ANS showing the distribution of juvenile Neoproterozoic crust and adjacent regions of Archean–Mesoproterozoic crust (after Fritz et al.^35^ and Johnson^87^ and Cenozoic plate boundaries, Red Sea-Gulf of Aden spreading centers, and the East African Rift system. CED, Central Eastern Desert; NED, North Eastern Desert; SED, South Eastern Desert; SES, South Ethiopian Shield; WES, West Ethiopian Shield, (**b**) Geological map of the Egyptian Nubian Shield. (modified from Johnson et al.^34^) showing location of the N-S shear zones. The figures were created by ArcGIS Desktop v. 10.8. https://www.esri.com/en-us/arcgis/products/arcgis-desktop/overview, and SmartSketch v. 4.0 software; https://smartsketch.software.informer.com/4.0/).

Since the pivotal research conducted by Abdelsalam and Stern^20^, there has been a growing interest among researchers in examining sutures and shear zones within the Arabian-Nubian Shield (ANS) and likely throughout the entire East African Orogen (EAO; Fig. 1a). Various types of deformational belts have been identified in the ANS (Fig. 1b). The first category is associated with arc-arc and arc-continental sutures, while the second encompasses post-accretionary structures, which consist of north-trending shortening zones and northwest-trending strike-slip faults. The authors suggested that arc-arc sutures indicate a collision between arc terranes that occurred approximately 800 to 700 Ma. In contrast, the arc-continental sutures delineate the eastern and western boundaries of the ANS, characterized by north-trending deformational belts that formed when the ANS collided with eastern and western Gondwana around 750 to 650 Ma^11,21^. The post-accretionary structures formed between roughly 650 and 550 Ma due to continued shortening within the ANS. The Egyptian Nubian Shield is marked by numerous shear zones that extend for tens to hundreds of kilometers, oriented in NW-SE, N-S, and NE-SW directions across the Central and Southern Eastern Desert of Egypt.

Shear zones in the ENS are classified into two main groups: syn-accretion shear zones and post-accretion shear zones. The NNE-oriented Hamisana Shear Zone belongs to the syn-accretion group. In contrast, the post-accretion group includes NW-trending Najd-related Shear Zones, such as the Atalla, Nugrus, and Hodein-Karite shear zones (Fig. 1b). This group also covers the relatively younger ENE- to E-trending shear zones and shear belts, including the Mubarak-Barramiya Shear Belt^21–29^. Additionally, the N-S-trending shear zones identified in this study are linked to the post-accretion group. Exploring their relationship to the NW- and NE-trending shear zones is one of the main goals of this study.

Neoproterozoic strike-slip shear zones, extending hundreds of kilometers and reaching up to 10 km in width, are prominently visible in the ENS. These zones represent the continuation of the Najd Fault System from the Arabian Shield to the Nubian Shield and are characterized by interconnecting strike-slip shear zones and transpressional belts. Numerous studies have investigated the strike-slip shear zones that run NW-SE and NE-SW^14–16,26–38^. Nevertheless, the N-S shear zones have not been examined as thoroughly. Notably, Abd El-Wahed et al.^6^ were the first to document one of these N-S shear zones in the Central Eastern Desert of Egypt, stretching from Wadi Safaga in the north to Gabal El Shalul in the south. The N-S Shear Zone, dating from 600 to 590 Ma, is a prominent geological structure within the Egyptian Nubian Shield, extending more than 140 km and attaining widths of up to 10 kilometers^6^. This shear zone is distinguished by conjugate shear zones that formed during the Najd Orogeny. The primary shear plane is oriented NW-SE, while additional shear zones that trend NE-SW and N-S function as conjugate structures^6,7^.

Integrating geophysical, structural, remote sensing (RS), and field data is significant for accurately delineating fault systems and shear zones, which often serve as key controls on hydrothermal fluid flow and mineralization. RS techniques are essential for delineating lithological contrasts and alteration zones, while geophysical strategies, particularly potential-field surveys, support the inference of subsurface structures and the delineation of fault kinematics and crustal architecture^26,27,39–50^. Structural analysis derived from structural mapping and field-based kinematic indicators introduces influential constraints on the deformation history, fault orientation, and tectonic regime. When integrated, these methods and datasets promote the detection of mineralized shear zones and structures with heightened spatial precision. For instance, in the Egyptian Eastern Desert, the combination of aeromagnetic and RS data with field verification has successfully recognized mineralization-bearing shear zones^49,51^. These investigations highlight that a multidisciplinary procedure significantly improves the targeting of mineral deposits associated with shear zones and structurally controlled areas.

This study is the first to examine the geometry of large N-S shear zones in the ENS (Fig. 1b). This study uses data from aeromagnetic surveys, remote sensing, and field investigations along these shear zones. Analysis of the geometric and kinematic features of N-S-striking structures reveals the presence of critical transpressional systems (e.g., shear zones, flower structures, en echelon folds). This analysis demonstrates the importance of strain partitioning in the deformation of N-S shear zones. The identified structures and their potential connections to other tectonic features are understood in the context of the regional crust. To fully describe the longest N-S mega shear zones in the ENS, this study looks at their geometry and structural features, as well as how they connect to the NW- and NE-striking shear zones of the Najd Fault system and the E-W-striking shear zone of the Allaqi-Hienai shear belt.

## Geology of the Egyptian Nubian Shield

The Egyptian Nubian Shield (ENS) is the northeastern extension of the EAO (Fig. 1a), which is recognized as the largest continuous Neoproterozoic-Cambrian orogeny on the planet. This orogeny is divided into two main sections: the Arabian-Nubian Shield (ANS) in the north and the Mozambique Belt (MB) in the south^35,52,53^. During the Neoproterozoic era, the supercontinent Rodinia was formed through the convergence of East and West Gondwana along the Pan-African Mozambique Belt. The break-up of Rodinia is believed to have commenced around 870-750 Ma^54,55^. The Nubian Shield is categorized into three key stages: the development of arc terrains, the accretion of the arc to the Nile Craton, and the post-accretion reworking of the arc^20,30,56^. The ENS covers an area of approximately 100,000 km², primarily located in the Red Sea hills of the Eastern Desert and southern Sinai^57^, with minor extensions into the south of the Western Desert (Fig. 1a). The ENS basement complex is made up of four central tectonostratigraphic units: high-grade gneisses and migmatites; volcanic and volcanosedimentary units that formed on an arc; dismembered ophiolites; and the Hammamat and Dokhan supracrustal sequences from the Ediacaran period, along with granitoids^36^. Sheared and ductilely deformed granitoid gneisses are found in antiformal “domal” structures, typically encircled by low-grade supracrustal assemblages^6,7,10–12,14–16,30,31,36–38,58,59^. Stern and Hedge^61^ categorize the Eastern Desert of Egypt into three distinct domains: North, Central, and South Eastern Deserts (NED, CED, and SED).

The CED is the best-known of the three subdivisions of the Eastern Desert, primarily for its intriguing and informative supracrustal sequences. This region predominantly comprises Cryogenian and Tonian-age rocks, which are divided into two distinct tectonostratigraphic units: the structural basement (lower tier) and the structural cover (upper tier). The structural basement, or lower unit, consists of high-grade metamorphic gneisses, migmatites, schists, and amphibolites. In contrast, the structural cover, known as the Pan-African nappes, features low-grade metamorphosed ophiolite slices and arc metavolcanics^62^. Ophiolitic rocks, arc metavolcanics, and volcanoclastic metasediments dominate the CED. Based on U-Pb zircon ages of about 750 Ma, these suprasubduction zone ophiolite assemblages are the oldest known units in the CED^53,63,64^. The Dokhan volcanic suite emerged during the later phases of crustal development within the CED. This is linked to the formation of molasses-type Hammamat sediments^33^. The CED is distinguished by an NW-trending tectonic structure that delineates the NW-SE sinistral shear zone of the NFS. The deformation events within the CED of Egypt can be categorized as follows^60^: (i) D1, associated with NNW-directed thrusts; (ii) D2, linked to thrusts directed towards the northeast and southwest; (iii) D3, which is connected to sinistral movements along the northwest-trending shear zones of the NFS; (iv) D4, related to dextral movements along northeast-trending shear zones; and (v) D5, representing subsequent events. The tectonic environment transitioned from a compressional arc accretion framework to a sinistral transpressional regime during the period between 650 and 540 Ma^12,14–16,30,36,60,65–67^.

The Neoproterozoic geological events in the SED remain inadequately characterized, as this region has received limited research attention. It is distinguished by the absence of distinctive sedimentary sequences, such as banded iron formations (BIFs) or diamictites, but it does contain massive sulfide deposits formed in volcanoes. Furthermore, the SED lacks significant Ediacaran sedimentary or volcanic sequences and exhibits serpentinite proportions comparable to those found in the CED^68,69^.

The NED is characterized by its unique composition of Ediacaran igneous rocks, which sets it apart from the CED and SED. It lacks ophiolites, has unidentified BIF and diamictite, shows no evidence of Najd deformation, and features a significant presence of dike swarms, epizonal A-type granites, and Dokhan Volcanics^70^.

### Large-scale shear zones in the Egyptian Nubian Shield

The Egyptian Nubian Shield is divided into three main domains^14,67^. These zones are (i) the Southern Compressional Domain, (ii) the Central Transpressional Domain, and (iii) the Northern Extensional Domain (Fig. 1b). The Southern Compressional Zone has features associated with arc-arc sutures and shear zones that formed during accretion^20^. These include the Hamazana shear zone and the Allaqi-Heiani-Onib-Sol Hamed suture. The Central Transpressional Zone is bordered to the south by the Kharit-Hodein shear zone and to the north by the Queih shear zone. This zone is mainly affected by deformational zones associated with the Najd Fault System (NFS), which formed after accretion. It is characterized by NW-trending sinistral strike-slip shear zones (e.g., Kharit-Hodein, Nugrus, Atalla, Queih) and fold structures, along with NE-SW dextral shear zones like the Mubarak-Baramyia and Barud shear belts. The Northern Extensional Zone is bounded to the south by the Barud shear belt and is characterized by extensional structures, including the Dokhan Volcanics and post-tectonic granites. Table 1 summarizes major structures, deformation events, structural styles, orientations, and ages, major shear zones, and post-accretionary structures in the ENS.

**Table 1:** Major structures, deformation events, structural styles, orientations and ages, major shear zones and post-accretionary structures in the Egyptian Nubian Shield.

Major structure	Deformation event	Structure style	Shear belt	Orientation	Age of deformation (Ma)	Developed structures	References
Sutures	Arc–Arc suturing. (D1)	Associated with E- or W-verging ophiolitic nappes, deformed by upright folds and strike-slip faults related to oblique collision	Allaqi-Heiani-Gerf-Onib-Sol Hamed-Yanbu suture system	E to NE	Formed during terrane accretion, between 800 and 700 MA	Characterized by N- or S-verging ophiolitic nappes that were later steepened by upright folds. NNE-dipping thrusts and SSW-vergent folds from N-S to NNE-SSW shortening	Abdelsalam and Stern, 1996^20^; Kusky and Ramadan, 2002^72^; Abdelsalam et al., 2003^71^
Syn-accretionary structures	E-W Shortening Zones (D2)	These structures developed between approximately 660 and 610 Ma due to continued regional shortening of the ANS after the main terrane accretion phases.	Hamisana shear zone	N-S	660–610	Early N-trending upright folds. Late NE-trending dextral strike-slip faults	Stern et al., 1990^74^; de Wall et al. 2001^75^
Post-accretionary structures	NE-SW shortening and NW-trending shear zones (D3)	Developed later, between approximately 630–530 Ma, representing the culmination of the shortening deformation	(i) Kharit-Hodein, (ii) Queih, (iii) Nugrus, (iv) Atallah	NW-SE NNW-SSE	630–530	Early dextral strike-slip faults & shear zones Late transpressional sinistral strike-slip faults & shear zones	Moore, 1979^77^; Stern, 1985^78^; Johnson et al., 2011^34^; Fritz et al., 2013^35^; Hamimi and Fowler, 2021^66^; Abd El-Wahed et al., 2022; 2023^80^; 2024^37,38^; Abd El-Wahed and Attia, 2022^14^; 2023^15^, 2025^16^
	E-W shortening and N-S-trending shear zones (D4)		(i) Safaga-Shalul (ii) Wadi Kareim-Umm Bisilla (iii) Um Gheig-Nugrus (iv) Barramiya-Mueilha (v) Abu Swayel- Muqsim (vi) Himitrah-Madari	N-S	630–530	Major dextral strike-slip faults and shear zones, which often truncate or rework older structures. These systems can be transpressional, combining strike-slip movement with thrusting and folding	Abd El-Wahed et al., 2025^16^
	E-W shortening and NE-trending shear zones (D5)		(i) Mubarak-Baramyia (ii) Barud	NE-SW ENE-WSW	630–530	Transpressional dextral strike-slip shear zones, thrusts and folds	Greiling et al., 1994^86^; Kamal El-Din and Abdelkareem, 2018; Abd El-Wahed, 2014^81^

#### Allaqi-Heiani suture (AHS)

The Allaqi-Heiani Suture (AHS) extends over 200 km from Gabal Um Shilman in the west to Nasser Lake, reaching the N-S-trending Hamisana shear zone in the east. This geological feature is prominent in satellite images and aerial photomosaics of the Gabgaba-Elba Topographic Sheets and exhibits orientations ranging from east-west to NW-SE to N-S. These orientations are perpendicular to the Wadi Allaqi to the west and align with its southern side to the east^6,7,14,20,71,72^. Tectonic interactions along the AHS (Table 1) are characterized by folding, thrusting, and significant imbrication of the Gabal Gerf Nappe over the Gabgaba arc terrane, dating back to 830-720 Ma. Research on ophiolitic formations within the Gerf Nappe indicates a predominance of large ophiolite blocks and steep thrust slices, with limited mélange^20^.

Abdelsalam and Stern^20^ identified four Neoproterozoic deformation phases (D1-D4) that shaped the AHS. Phases D1 and D2 correspond to the early collision of the Gerf terrane with the Haya and Gabgaba terranes, while D3 and D4 pertain to subsequent collisional stages. The AHS displays a sinistral shear sense, indicated by shear band mylonitic foliation and S-C fabrics^6,7,14,69,73,74^. The shearing has resulted in a complex folding history with planar and non-planar refolded sheath folds. Structural analyses reveal a polyphase deformation history, notably two significant events: Event D1, involving N-S to NNE-SSW shortening, and Event D2, marked by ENE-WSW shortening^73,74^.

#### Hamisana Shear Zone (ASZ)

The ANS (Table 1) is mainly composed of N-S-oriented zones of highly foliated, isoclinally folded rocks^34^, such as the Hamisana Shear Zone (HSZ) and the Oko Shear Zone (OSZ). These zones show how E-Gondwana and W-Gondwana interacted. The HSZ covers about 15,000 km² and extends across southeastern Egypt and northeastern Sudan. It is composed of gneissic and schistose rocks, as well as folded ophiolite fragments from the Allaqi-Heiani and Onib-Sol Hamed sutures^20,75,76^. This shear zone significantly influences the movement of the AHS and extends northward, delineating the Gerf Nappe to the east (Fig. 1b). After 660 Ma, the HSZ started to change shape. It shortened significantly from east to west and grew longer from north to south. At the same time, it underwent metamorphic changes from greenschist to amphibolite, resulting in upright isoclinal folds and vertical foliation^75^. This deformation likely concluded around 550 Ma. The dominant ductile deformation in the HSZ is marked by nearly coaxial folding around an N-S axis, reflecting a shortening direction perpendicular to the zone, specifically east-west^75^.

#### The Najd Fault System (NFS)

The NFS (Table 1; Fig. 1b) is an extensive network of NW-SE sinistral strike-slip faults and shear zones within the ANS. It is among the largest Neoproterozoic shear zones worldwide^6,14–16,34,35,38,66,77–80^. The NFS (620-540 Ma) is approximately 400 km wide and 1100 km long, with subsurface extensions that may reach up to 2000 km (76). The system was reactivated by the Tertiary Red Sea rifts^76,79^. Initially considered post-accretionary, the NFS features are linked to the escape tectonics associated with the Gondwana collision^20,71^. Structures in the NFS formed under sinistral transpressional deformation, with a central compressional axis oriented to the NW of the boundary^81^.

The NFS plays a crucial role in the development and subsequent exposure of gneiss-cored domes (Fig. 1b) and Hammamat molasse deposits found within the Egyptian Nubian Shield^10,30,32,34,61,65,66,82–84^. Gneiss domes in the ENS are compared to those in other tectonic settings, such as those in the Zagros hinterland-fold-and-thrust belt^5,6^. Furthermore, the NW–SE oriented strike-slip faults that surround different gneiss core complexes in the Nubian Shield show breaks, with the strain decreasing significantly as one moves away from the gneiss domes^37^. These include Sibai, Meatiq, Shalul, and Hafafit. The primary offset direction along the NFS is sinistral^30,76^. However, some researchers have suggested that an initial dextral phase may have preceded the sinistral movement^76^.

The sinistral shear zone of the ENS (Fig. 1b) in the CED and the northern parts of the SED is characterized by a dominant NW-trending structural fabric^14,21,30,31^. A multitude of studies indicate that the D2 deformation event within the central area of the ENS was associated with lateral extension and oblique convergence transpression, which ultimately led to the exhumation of core complexes during orogen-parallel extension approximately between 620 and 580 Ma^4,6,7,14–16,21–23,30–32,36,58,65,84^.

#### The Mubarak-Barramiya Shear Belt (MBSB)

The Mubarak-Barramiya shear belt (Table 1; Fig. 1b) was initially recognized by Abd El-Wahed and Kamh in 2010, who characterized it as a prominent high-strain zone extending from the Wadi Mubarak area along the Red Sea coastline to the region west of Barramiya. This shear belt is notable for its sheared ophiolite fragments embedded within a schistose mélange^81^. It serves as a demarcation line, separating the metavolcanic and volcanoclastic metasedimentary rocks to the north from the gneissic formations of the Hafafit dome. The different structural forms in this shear belt display a pattern of oblique convergence, resulting in transpression defined by simple shear. This mechanism results in the formation of a prominent flower structure located between Wadi Mubarak and the Hafafit dome. This flower structure spans the entire width of the CED (60). Abd El-Wahed^81^ noted that the NE-trending transpressional deformation heavily influences the formations associated with the NW-sinistral shear zones in the Mubarak-Barramiya shear belt.

#### The Qena-Safaga shear zone (QSSZ)

The Qena-Safaga shear zone (Fig. 1b) is identified as one of the most essential shear zones in the CED. For the most part, this zone trends NE-SW. It is divided into sections by significant subvertical strike-slip faults that strike in different directions, such as N-S, NE-SW, E-W, and NW-SE^85^. It serves as a boundary between the central and northeastern regions of the Eastern Desert^86^. The area is also characterized by NE-trending fractures and a series of oblique thrusts, which exhibit a northeast dextral component^62^.

## Methodology

### Remote sensing

Litho-structural mapping has effectively utilized Landsat-8 imagery, particularly in arid environments^88,89^. In the current study, two cloud-free Landsat-8 OLI/TIRS Level-1T images were used to generate structural maps and improve the delineation of geological contacts within the study area. These images were sourced from the U.S. Geological Survey Earth Resources Observation and Science Center (EROS) (http://earthexplorer.usgs.gov). The Landsat-8 data facilitated the identification of significant structural features and the differentiation of various lithologic units. The findings were subsequently integrated with aeromagnetic data and field observations. Launched in February 2013, the Landsat-8 sensor captures data across 11 spectral bands: nine bands cover the visible and near-infrared (VNIR) and short-wave infrared (SWIR) ranges (bands 1 to 9), while bands 10 and 11 cover the long-wave thermal ranges. This sensor is classified as providing medium spatial resolution, with 15 m for the panchromatic band, 30 m for bands 1-7, and 100 m for the thermal bands^90^. The spatial resolution aligns with the litho-structural mapping across extensive regions, including the study area. Both images were geometrically corrected to conform to the Universal Transverse Mercator (UTM) projection (Zone 36 N) and the World Geodetic System (WGS) 84 datum and ellipsoid. Image processing was conducted utilizing ENVI 5.3 (ENVI^®^ image processing and analysis software from ITT Visual Information Solutions) and ArcGIS 10.5 (from ESRI^®^ Environmental Systems Research Institute). Atmospheric correction was also implemented as a crucial step in pre-processing to mitigate atmospheric effects on the images^91^. This correction was performed using the Fast Line-of-Sight Atmospheric Analysis of Hypercubes (FLAASH) module integrated within ENVI. The two scenes from Landsat-8 imagery were mosaicked and cropped to fit the study area boundaries. The Enhanced Lee filter was also applied to the first principal component to refine the image for lineament detection further.

### Gravity data

**Fig. 2:**
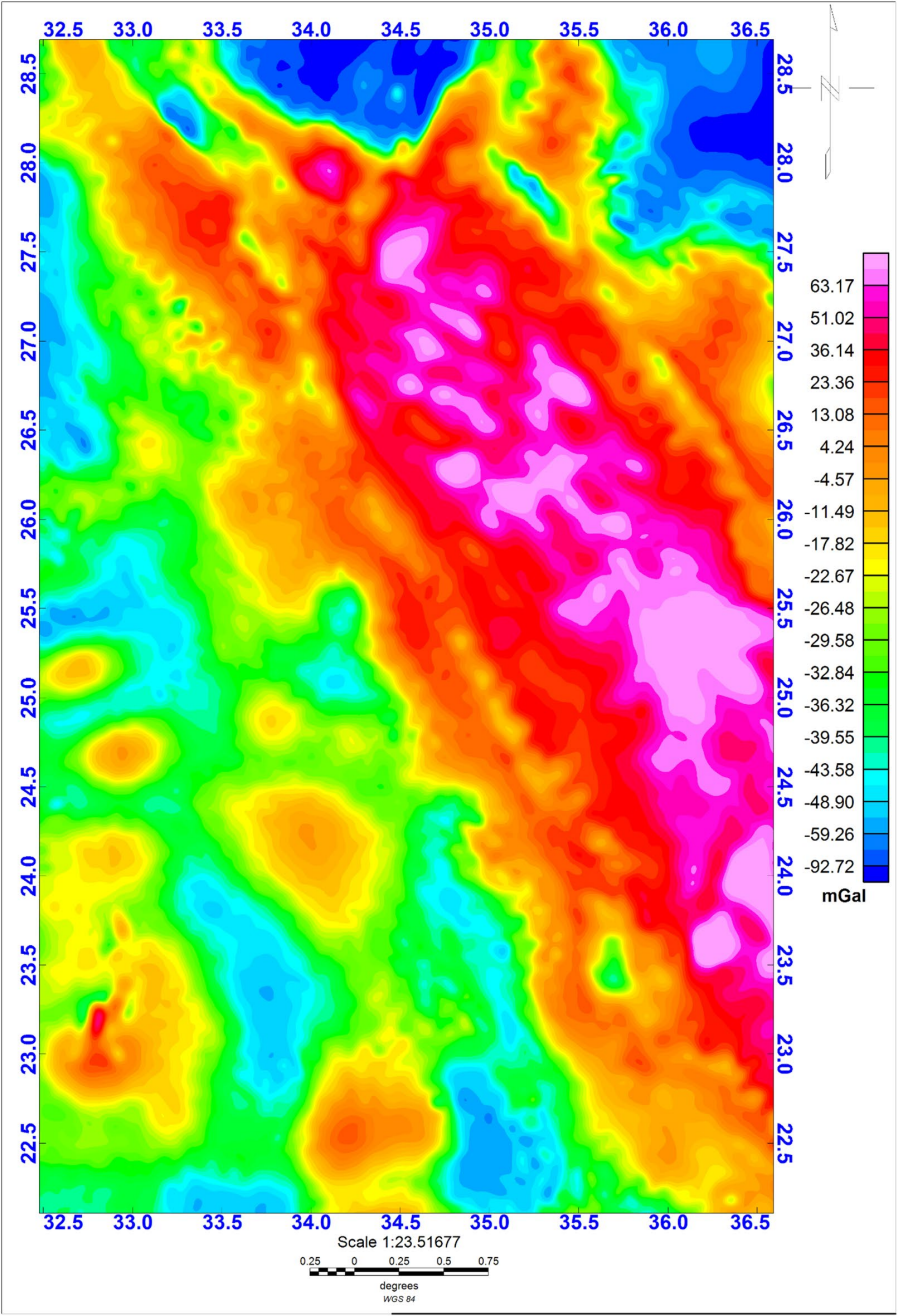
The geophysical Bouguer gravity (GBG) map of the Egyptian Nubian Shield (92). The figure was created by Geosoft Oasis montaj v. 8.3.3. (https://www.seequent.com/products-solutions/oasis-montaj/).

The geophysical Bouguer gravity (GBG) data utilized in our investigation are obtained from the World Gravity Model (WGM) 2012^92^. The geophysical Bouguer gravity (GBG) data utilized in our investigation are obtained from the World Gravity Model (WGM) 2012^92^. The GBG data used in this analysis originate from the global compilation developed from the Earth Geopotential Model 2008 (EGM2008), which was made publicly available by the Bureau Gravimétrique International (BGI). The original EGM2008 representative was produced by integrating a comprehensive spectrum of gravity observations, including marine surveys, terrestrial measurements, satellite-derived data acquired from the GRACE mission, and airborne campaigns. To generate the Bouguer anomaly field used here, the WGM2012 framework was used, which integrates several updates, including the 1-arc-minute ETOPO1 global topographic model, the DTU10 global gravity solution, and refined Arctic gravity coverage. All reductions were referenced to the World Geodetic-System 1984 (WGS84), and a definitive crustal density of 2.67 g/cm³ was assumed for Bouguer corrections. The GBG map (Fig. 2) demonstrates anomalies at shorter and longer wavelengths. The long-wavelength anomalies are usually associated with wide and plunging density origins, while short-wavelength ones are generally driven from shallow and limited lateral extent density sources. The GBG map of the investigation area (Fig. 2) reveals a notable and significant increase in gravity values towards the east, which can be interpreted as the response of oceanic crust sites in the Red Sea^12,93,94^.

### Logistic Total Horizontal Gradient (LTHG) filter

The numerical basis for the applied LTHG enhancement^95,96^ for detecting edges (faults/contacts/lineaments) in GBG data in the study area is presented in this section. The LTHG approach is the ratio of the first vertical gradient to the total horizontal derivative (HD) gradient, which yields noticeable tiny and large-amplitude boundaries together. The purpose of including this method is to produce a sigmoidal curve with the numerical logistic function. Its S-shape is closely related to that of the arctangent equation, which is commonly used to detect edges and boundaries of potential fields^12,93,95–97^.

1 $$\:LTHG={\left[1+exp\left\{-\:\frac{\frac{\partial\:THG}{\partial\:z}}{\sqrt{{\left(\frac{\partial\:THG}{\partial\:x}\right)}^{2}+{\left(\frac{\partial\:THG}{\partial\:y}\right)}^{2}}}\right\}\right]}^{-\alpha\:}$$

2 $$\:where,\:THG=\sqrt{{\left(\frac{\partial\:J}{\partial\:x}\right)}^{2}+{\left(\frac{\partial\:J}{\partial\:y}\right)}^{2}}$$

where, J is the field, $\:(\partial\:J/\partial\:x)$ and $\:(\partial\:J/\partial\:y)$ are the horizontal derivatives of the field in x and y directions.

The positive constant (α) governs the potential of *LTHG* and is expressed by the investigator. Experimental results on artificial models indicate that optimal performance was typically observed for *α* = 2–10. *THG* is the total horizontal amplitude^98^, $\:\partial\:z$ is the vertical derivative, and $\:\partial\:x$ and $\:\partial\:y$ are the horizontal components in *x* and *y* directions of the field.

### Field studies

In addition to creating new geological and structural maps of the initially identified north-south shear zones, field studies and detailed structural analyses have been carried out to clarify the structural features associated with them. The shear zones have been extensively studied through multiple traverses to document their geometry and movement. During each traverse, field observations, overprinting relationships, and structural measurements of orientation data were collected from specific observation stations. The orientations of the data and the oriented rock samples were recorded using a Brunton Compass. Compasses and GPS devices were employed to accurately map the orientation, shape, and distribution of the shear zones. Rock samples were collected from both the shear zones and the surrounding areas for later laboratory analysis. Thin sections of the oriented samples were prepared for examination. Analyzing the geometry and kinematics of shear zones requires careful examination of the orientations of fabrics and structures within the rocks, which is essential for understanding their shearing behavior.

## Results

### Regional Tectonic Fabric and Structural Trends from GBG data analysis

**Fig. 3:**
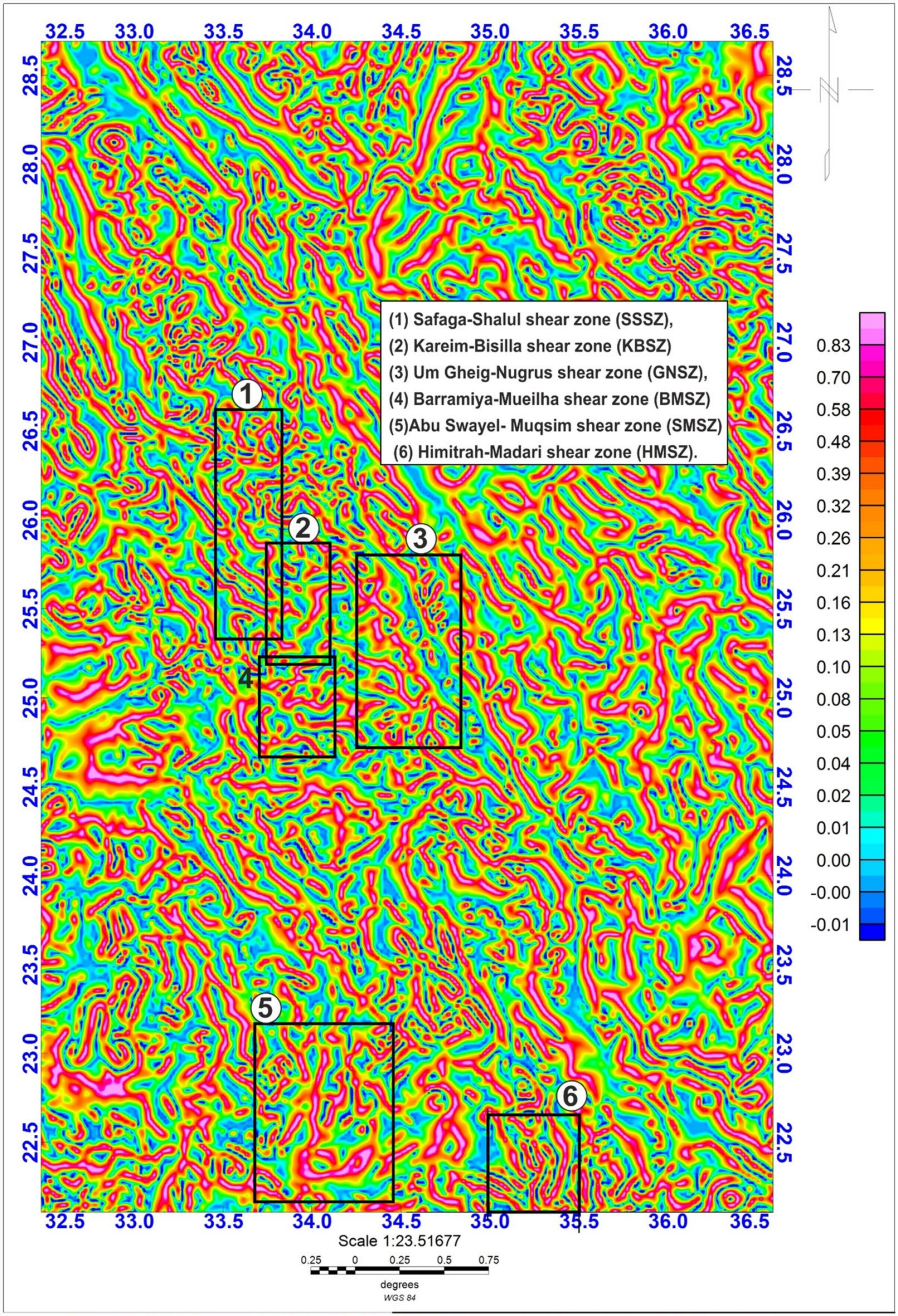
The LTHG map of the GBG data. The figure was created by Geosoft Oasis montaj v. 8.3.3. (https://www.seequent.com/products-solutions/oasis-montaj/).

The Logistic Total Horizontal Gradient (LTHG) and Local Tilt Horizontal Gradient (LTHG) filters are routinely used for mapping edges, faults, and contacts in potential field data, such as gravity or magnetic anomalies, by enhancing linear^12,96,97^. The LTHG filter, applied via Oasis Montaj to a high-resolution GBG gravity dataset (1 km grid spacing), enhanced linear features such as faults and contacts, revealing a dominant N-S trending fault system in the structural map (Fig. 3). The structural understanding of the LTHG results (Fig. 3) demonstrates a well-recognized system of prevalent N-S trending lineaments. The most notable of these directions aligns with line 34°E, extending longitudinally from 22°N to 27.5°N. This linear N-S feature includes a significant shear zone, promoting a prolonged reactivation along the weakness zone.

In the southern compressional domain (SCD) of the studied province (22°N–24.5°N), the prevailing orientation is E–W, which seems to be overprinted by later WNW to NW- and N-S trending structures. This overprinting framework illustrates the effects of successive deformational phases on the area’s structural development. At about 24.5°N, the fault and lineament patterns become more complex. Here, a set of E-W-oriented structures is notably recognized, dispersing throughout the region (Fig. 3). This localized direction presumably indicates a transient stress domain or tectono-magmatic interchanges that controlled the evolution of these components, conceivably associated with sideways regional extension or shearing^12,50,99^.

The Central Transpressional Domain (CTD) of the region (about 24.5°N–26°N) is characterized by a diverse assemblage of structural directions, mainly oriented NW, WNW, N–S, and NE (Fig. 3). The co-occurrence of these considerable trends indicates an area of deformation partitioning or structural interference^12,50,99^.

In contrast, the Northern Extension Domain (NED) of the investigation area (26°N–27.5°N) is controlled by NE- and N–S-trending structural features, with a subordinate existence of NW directions (Fig. 3). This structural regime indicates a potential transformation in the regional stress pattern, where NE and N–S directions become more prominent^12,50,99^.

### Geometry of the N-S shear zones in the ENS

The N-S shear zones are part of a larger system of shear zones that formed during the Neoproterozoic era and are explicitly associated with the Najd Fault System. These zones exhibit dextral (right-lateral) movement and are vital for understanding the tectonic framework of the Arabian-Nubian Shield, a key component of the East African orogenic belt. Extending over considerable distances, the N-S shear zones feature intricate structural geometries, including mylonitic schists and other metamorphic rocks. Their formation involved both pure and straightforward shear elements, reflecting a transpressive deformation regime that has shaped the region’s geological history. Additionally, the N-S shear zones interact with other structural elements, such as NW-SE- and NE-SW-trending shear zones, resulting in a complex network of deformation.

This study documents several large-scale N-S-trending shear zones within the Egyptian Nubian Shield and introduces new names for them. These shear zones are recognized based on their geographic extent and characteristic structural signatures, thereby providing a finer-scale understanding of the tectonic development of the Nubian Shield. These shear zones are named for the locations where they begin and end. These include: (i) Safaga-Shalul shear zone (SSSZ), (ii) Wadi Kareim-Umm Bisilla shear zone (KBSZ), (iii) Um Gheig-Nugrus shear zone (GNSZ), (iv) Barramiya-Mueilha shear zone (BMSZ), (v) Abu Swayel-Muqsim shear zone (SMSZ), and (vi) Himitrah-Madari shear zone (HMSZ). Table 2 summarizes the geometry, deformation history, and kinematics of the newly identified N-S shear zones.

**Table 2:** Geometry, deformation history and kinematics of the N-S shear zones.

Shear zone	Lithology	Geometry	Structures
Safaga-Shalul shear zone (SSSZ)	Gneisses, serpentinites, metabasalts, metagabbros, volcaniclastic metasediments, arc metavolcanics, a metagabbro-diorite complex, synorogenic granitoids, Dokhan Volcanics, Hammamat sediments, and late-to-post-orogenic granites.	140 km in length and attains widths of up to 10 km	E-W foliation (S1) in ophiolitic rocks and metasediments, Abu Fanani schist, El-Zarga metasediments -The NW-SE shear foliation (S2) prevails in Wadi Semna, Wadi Homeiriyia, and Wadi Atalla. -An imbricate thrust sheet that dips to the southwest and is oriented NW-SE. Folding in the Abu Marawat metavolcanics. - Serpentinites at Gabal Rubshi and Wadi El Saqia have a N-S orientation, aligned with the SSSZ. - Imbricate thrust sheets consist of serpentinite and volcanoclastic metasediments, with significant deformation producing mylonites and ultramylonites. - A lens of sheared metabasalt is found within talc carbonate serpentinite. - N-S-oriented thrust sheets of metavolcanic and metagabbro are thrust over serpentinite, intruded by Fawakhir granitoids. - An N-S thrust fault separates the metasediments from Shalul gneisses, which exhibit westward movement.
Wadi Kareim-Umm Bisilla shear zone (KBSZ)	serpentinite, metagabbro, metavolcanics, metasediments, Hammamat molasse sediments, and multiple types of granite. Post-Hammamat volcanic formations, including Felsites and trachytic plugs	The N-S shear fabric marks the KBSZ boundaries, extending about 70 km from Gabal Umm Salatit to Wadi Karein	-The Wadi Kareim basin is a significant east-west-oriented syncline. -F2 folds mainly run NW-SE. -S2 represents an axial planar foliation associated with F2 folds. - F2 folds are characterized by both overturned and tightly plunging mesoscopic structures. - NW-trending imbricated thrust sheets. - S3 foliation reveals that the KBSZ consists of north-trending, sub-parallel, and anastomosing lineaments. - S3 foliation strikes from N-S to NNE-SSE and typically show subvertical or steep dips towards the NE or SW. - A series of N-S trending thrusts has developed north of Wadi Zeidun—these imbricate thrust sheets, composed of metavolcanics and volcanoclastic metasediments. - The F3 folds mainly trend N-S and NNE-SSW, with a predominant plunge toward the north.
Um Gheig-Nugrus shear zone (GNSZ)	Gneisses, ophiolitic associations, volcaniclastics, metavolcanics, Metagabbros, Hammamat sediments, syn- to post tectonic granites.	100 km in length and attains widths of up to 3–5 km, from Wadi Um Ghieg to Wadi Nugrus	-The GNSZ comprises a network of interconnecting ductile shear zones that trend N-S, NNW-SSE, and NNE-SSW. -The oldest foliation, designated as S1, trends E-W and has been modified by various deformation events, resulting in the formation of NW-SE (S2), N-S (S3), and NE-SW to ENE-WSW (S4) foliations, along with associated folds. -The N-S-striking subvertical S3 foliation, L3 stretching subhorizontal lineation, and F3 folding are distinctly visible in the volcanoclastic metasediments and sheared metavolcanics located along Wadi Umm Luseifa and at the entrance of Wadi Um Gheig. -F3 folds has developed within the volcanoclastic metasediments along the western bank of Wadi Wizr. -A notable change in the fabric direction from a NE-SW orientation to NNE-SSW north of Gabal Abu Karanish and south of Wadi Abu Dabbab. -NE-oriented fabric has been locally influenced by shearing that trends NNE-SSW between Wadi Dubr and Wadi Abu Dabbab, -The S3 fabric is overprinted by NE-trending dextral oblique shear zones, leading to the formation of an ENE-WSW-trending fabric (S4) and significant folds (F4) -The N-S-oriented fabric (S3) of the GNSZ extends from Shikh Salim through Wadi El Barda to the deflection at Wadi Nugrus, where it influences the NW-trending fabric and thrusts of Wadi Garf, Wadi Um Khariga, Sukari, and Wadi Ghadir
Barramiya-Mueilha shear zone (BMSZ)	Ophiolite slices, Metavolcanics, volcanoclastic metasediments, diorite complexes, late or post-orogenic intrusions	40 km in length and attains widths of up to 3 km, from Wadi Mueilha in the south to Wadi Beizah in the north	-D1 involves stacking of ophiolitic nappes -D3 highlights the development of a significant dextral shear zone with a palm-tree-like configuration. -D4 is marked by extensional structures indicating later brittle deformation. -The S1 foliation is characterized by ENE-striking shear features developed around the ophiolites, constrained by thrusts -Steeply dipping mineral foliation (S2), oriented NW-SE to WNW-ESE, is most prominently observed in schistose rocks and in sheared metagabbro, meta-agglomerate, and metatuffs -The Barramiya-Mueilha region is characterized by increased S2 schistosity and mylonitic foliation that trends northwest. During the D3 deformation phase, the Barramiya-Mueilha shear zone emerged as a significant dextral shear zone oriented NNE -This shear zone exhibits a palm-tree-like configuration, with the central plane oriented NNE-SSW and steeply dipping towards the WNW.
Abu Swayel- Muqsim shear zone (SMSZ)	Gneisses, dismembered ophiolites, metavolcanic-metasedimentary sequences from an island arc, and granitoid intrusions	70 km in length and attains widths of up to 24 km, from Wadi Bagharid and Gabal Shashoba in the east to Wadi Ungat, Gabal Murra, and Gabal Shilman in the west	- D1 includes pervasive cleavage foliation (S1), mineral stretching lineation (L1), folds oriented toward the east and west-northwest (F1), significant thrust faults trending E-W to WNW-ESE, and imbricate slices of ophiolite. - NW-plunging folds and NW-SE foliation (S2) overlay thrust fabrics in schistose metavolcanic and ophiolitic rocks, indicating D2 deformation. - The sinistral transpression along pre-existing WNW-oriented thrusts and numerous NW-SE shear zones is thought to stem from the ongoing tectonic movement of ophiolitic nappes from the southeast to the northwest. - S3 fabric along NNW-SSE thrust-dominated shear zones and thrust segments. D3 structures include tight to isoclinal folds trending NNW and N, and strike-slip faults that overlay older geological structures. F3 anticlinoria and synclinoria, ranging from meter to kilometer scales. The SMSZ is characterized by minor N-trending dextral zones that form a sigmoidal pattern with a mainly N-S orientation.
Himitrah-Madari shear zone (HMSZ)	Gneisses, ophiolitic nappes, island arc-related metasediment-metavolcanic, and syn- and post-orogenic intrusions	60 km in length and attains widths of up to 10 km, from Gabal Himitrah to Gabal Madari	-The shear zone is the northern extension of the Hamazana shear zone -D1 is E-W thrusting and folding (F1) that impacted ophiolitic and island-arc-related metasediments. -D2 features NW-trending structures. -D3 involves E-W contraction and the development of N-S-striking mylonitic foliation and N-trending folds, creating the Himitrah-Madari shear zone. -F1-folding events exhibit steeply dipping to vertical north-dipping and, less frequently, south-dipping planes. -F2 (NW–SE) folds are linked with NW-thrust faults in ophiolitic rocks and amphibolite -F3 (N–S) folds are widely observed in the HMSZ area, ranging from open at the kilometer scale to tight, upright, and slightly asymmetric.

#### Safaga-Shalul shear zone (SSSZ)

**Fig. 4:**
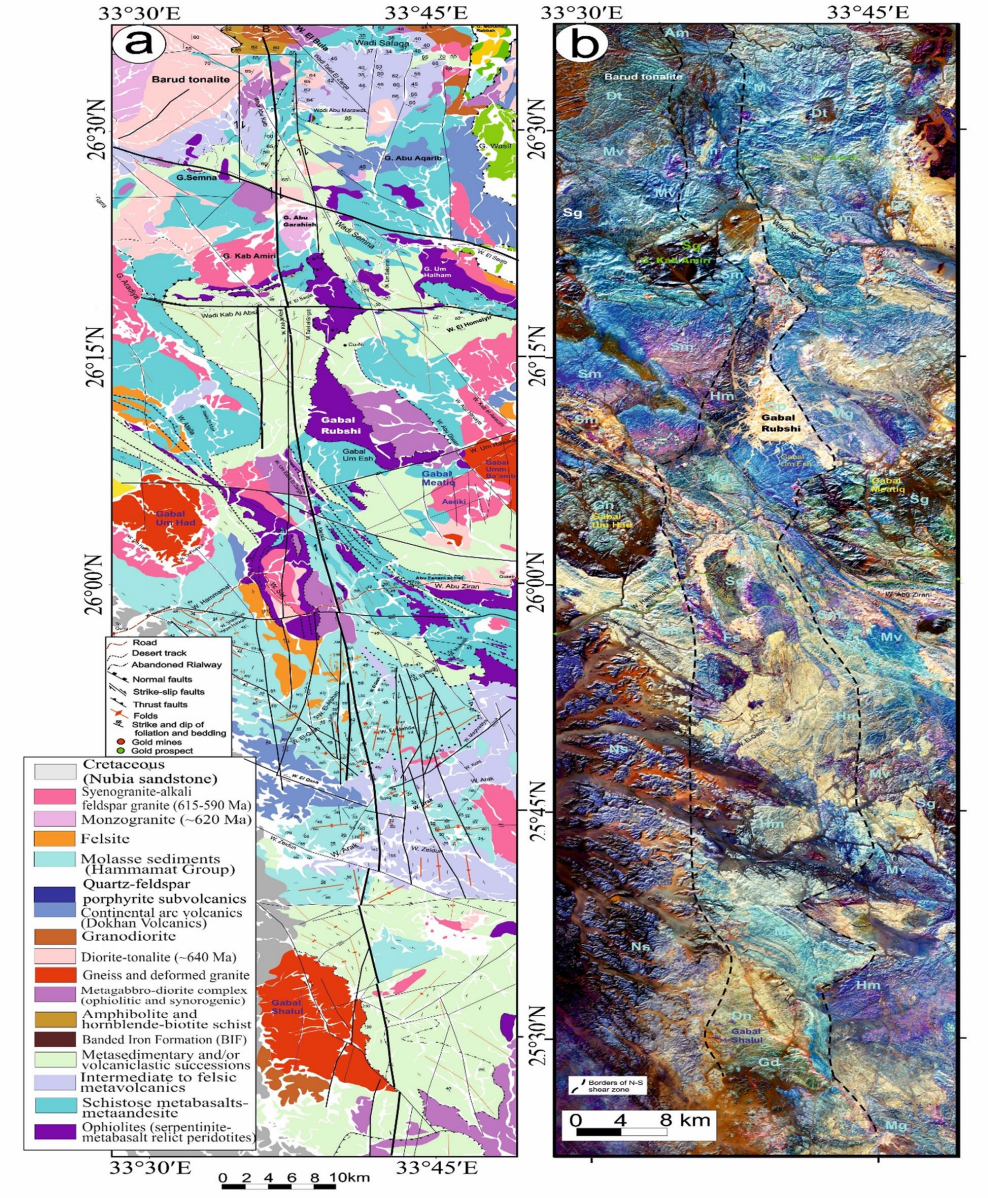
(**a**) Geological map of Safaga-Shalul shear zone (SSSZ) (modified after Abd El-Wahed et al.^6^). The figure was created using ArcGIS Desktop v. 10.8 (https://www.esri.com/en-us/arcgis/products/arcgis-desktop/overview) and SmartSketch v. 4.0 (https://smartsketch.software.informer.com/4.0/). (**b**) Band ratios of 4/7, 3/6, and 2/5 for the Safaga-Shalul shear zone. (The figure was created by ENVI v. 5.6.2. software; (https://www.l3harrisgeospatial.com/Software-Technology/ENVI).

**Fig. 5:**
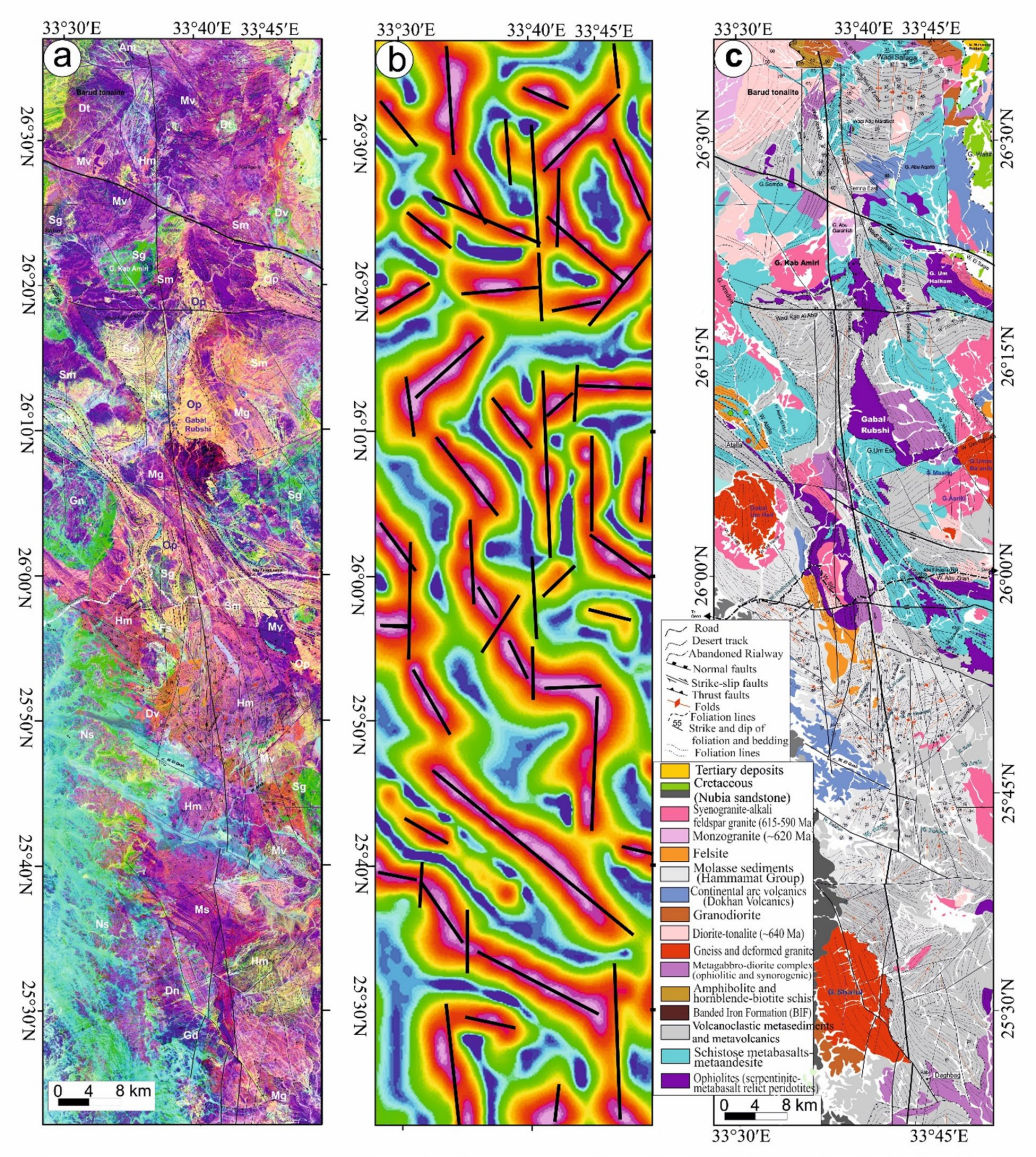
(**a**) Minimum Noise Fraction (MNF) 2, 3, and 7 in RGB showing the extent of the Safaga-Shalul shear zone (SSSZ). The figure was created by ENVI v. 5.6.2. software; (https://www.l3harrisgeospatial.com/Software-Technology/ENVI), (**b**) The Logistic Total Horizontal Gradient (LTHG) map of the SSSZ The figure was created by Geosoft Oasis montaj v. 8.3.3. https://www.seequent.com/products-solutions/oasis-montaj/). (**c**) Structural map of Safaga-Shalul shear zone (SSSZ) (modified after Abd El-Wahed et al.^6^). Note: Gn: Gneiss, Op: Ophiolite, Sm: Schistose metavolcanics, Mv: Metavolcanics, Ms: Metasedimentary, Am: Amphibolite, Mg: metagabbro-diorite, Dt: Diorite-tonalite, Gd: Granodiorite, Dv: Dokhan volcanics, Hm: Molasse sediments, Fs: Felsite, Pg: Monzogranites, Sg: Synogranites and Ns: Nubian sandstone. The figure was created using ArcGIS Desktop v.10.8. https://www.esri.com/en-us/arcgis/products/arcgis-desktop/overview, and SmartSketch v. 4.0 software; https://smartsketch.software.informer.com/4.0/).

This shear zone (Table 2), initially identified and described by Abd El-Wahed et al.^6^, spans over 140 km in length and attains widths of up to 10 km, stretching from Wadi Safaga in the north to Gabal Shalul in the south (Figs. 4a, b, and 5a). The region influenced by the SSSZ exhibits a diverse array of Neoproterozoic rock types, including gneisses, serpentinites, metabasalts, metagabbros, volcaniclastic metasediments, arc metavolcanics, a metagabbro-diorite complex, synorogenic granitoids, the Dokhan Volcanics, Hammamat sediments, and late-to-post-orogenic granites.

The ophiolitic suite encompassing Saqia, Sodmein, Fawakhir, Gabal El-Rubshi, and Kab Amiri consists of various fragments, including serpentinites, metabasalts, and mélange rocks. In Fawakhir, Rubshi, and Wadi Saqia, the serpentinite formations run north-south, following the SSSZ, and are thrusted over the volcanoclastic metasediments^6,100,101^. In Wadi Safaga, the volcaniclastic metasediments include metamudstones, metasiltstones, metagraywackes, meta-conglomerates, and schists^6,102^.

Arc metavolcanics occur in elongated bodies and exhibit a range of colors, including dark grey, greenish grey, and pink. The Dokhan Volcanics are prominently visible in the regions adjacent to Wadi Saqia, Wadi El-Qash, and Wadi Atalla^100,103^. In Wadi El Qash, the Dokhan volcanics comprise pyroclastic materials ranging from felsic to intermediate in composition. The Hammamat deposits are primarily located in Wadi Hammamat, El Qash, and Zeidun, with more minor occurrences found along Wadi Abu Kalb. The stratigraphic sequence is characterized by layers of alternating siltstone and mudstone, supported by polymictic conglomerates of varying sizes^33,104^.

The Meatiq core complex consists of metamorphic rocks, including the altered Um Banib gneissic granite, amphibolite-facies schists, and gneisses, which transition into mylonites along its margins within two northwest-oriented shear zones^30^. The granite covers approximately 100 km² and is mainly composed of altered monzogranite^105^. In the Wadi Um Had area, a northwest-trending gneissic dome is surrounded by schistose and mylonitic layers. The Shalul metamorphic core complex exhibits an open, NNW-SSE-oriented domal structure, primarily composed of Shalul monzogranite gneisses^14^. The Barud Tonalite, located in the northern SSSZ, shows discordance with amphibolite foliation and intersects with amphibolites and a metagabbro-diorite complex^6,106,107^. The oval-shaped Abu-Ziran granitoids are oriented WNW–ENE, nearly parallel to the foliation of the Abu Fannani schist^63^. The Fawakhir granitoids intrude into the Fawakhir ophiolite. At the same time, the Abu Gaharish pluton is rich in Nb, W, Ta, Sn, and U. The Arieki granitoids are found within the Meatiq dome.

False-color composites using RGB bands 4/7, 3/6, and 2/5 (Fig. 4b) improved visibility of contacts between rock units and structural features. Variations in color and foliation orientation highlight shear zones, thrust faults, and strike-slip faults, with N-S- and NW-SE-strike-slip faults indicated by displaced rock types. The MNF 237 in RGB (Fig. 5a) is most vivid, distinguishing rock units and structural styles: ophiolites in pale violet, metavolcanics in yellowish-orange, metasediments in cyan, and Hammamat and Dokhan volcanics in bluish-violet. Shear zones NW-SE and N-S are identifiable. The LTHG map of the SSSZ (Fig. 5b) shows dominant structural directions: N-S, NW-SE, WNW-ESE, NE-SW, and E-W.

The map of the Safaga-Shalul shear zone^6^ shows the oldest fabric as E-W foliation (S1) in ophiolitic rocks and metasediments (Fig. 5c). This E-W fabric appears in the Abu Fanani schist, El-Zarga metasediments, and intermediate metavolcanic units. The earliest S1 in the Meatiq dome features gneissic banding and schistosity in amphibolite enclaves of the Um Baanib granite^6^. The Abu Marawat metavolcanics have tabular zones with S1 foliations and steeply inclined lineations. Microstructural features in the mylonitic shear zones at the boundaries of metasediments, amphibolites, and metavolcanics show top-to-the-NW movement. These shear zones lie between the Bula granodiorite and Wadi Safaga (Fig. 5c).

The NW-SE shear foliation (S2) prevails in Wadi Semna, Wadi Homeiriyia, and Wadi Atalla. The rocks in Wadi Homeiriyia include serpentinites, schistose metabasalts, and volcanoclastic metasediments (Fig. 5c), forming an imbricate thrust sheet that dips to the southwest and is oriented NW-SE. The folding in the Abu Marawat metavolcanics corresponds with the axial-planar foliation (S2). These folds are generally open and gentle, with axial planes that are either upright or steeply inclined, oriented NW-SE. The fold axes exhibit a moderate plunge toward the southeast.

The Atalla shear zone (ASZ) traverses the western part of the Meatiq area and remains a subject of contention among researchers. Two opposing models have been put forward to elucidate its structure: one posits that the Atalla shear zone functions as a NW-SE strike-slip shear zone, whereas the other contends that the ductile fabrics observed in the folded rock formations are indicative of a subhorizontal shear zone characterized by a top-to-NW shearing orientation.

The thrust faults are situated south and west of the Meatiq dome (Fig. 5c) and display orientations ranging from NW-SE to NNW-SSE, with dips in both the NE and SW directions^6^. The SSSZ is a brittle-ductile shear zone marked by several episodes of brittle reactivation and a deformation gradient. This zone comprises mylonitic schist, metasediments, augen gneisses, and molasse sediments. It connects the northeastern Barud tonalite and the southern Shalul gneiss and is categorized into three distinct domains: northern, central, and southern.

**Fig. 6:**
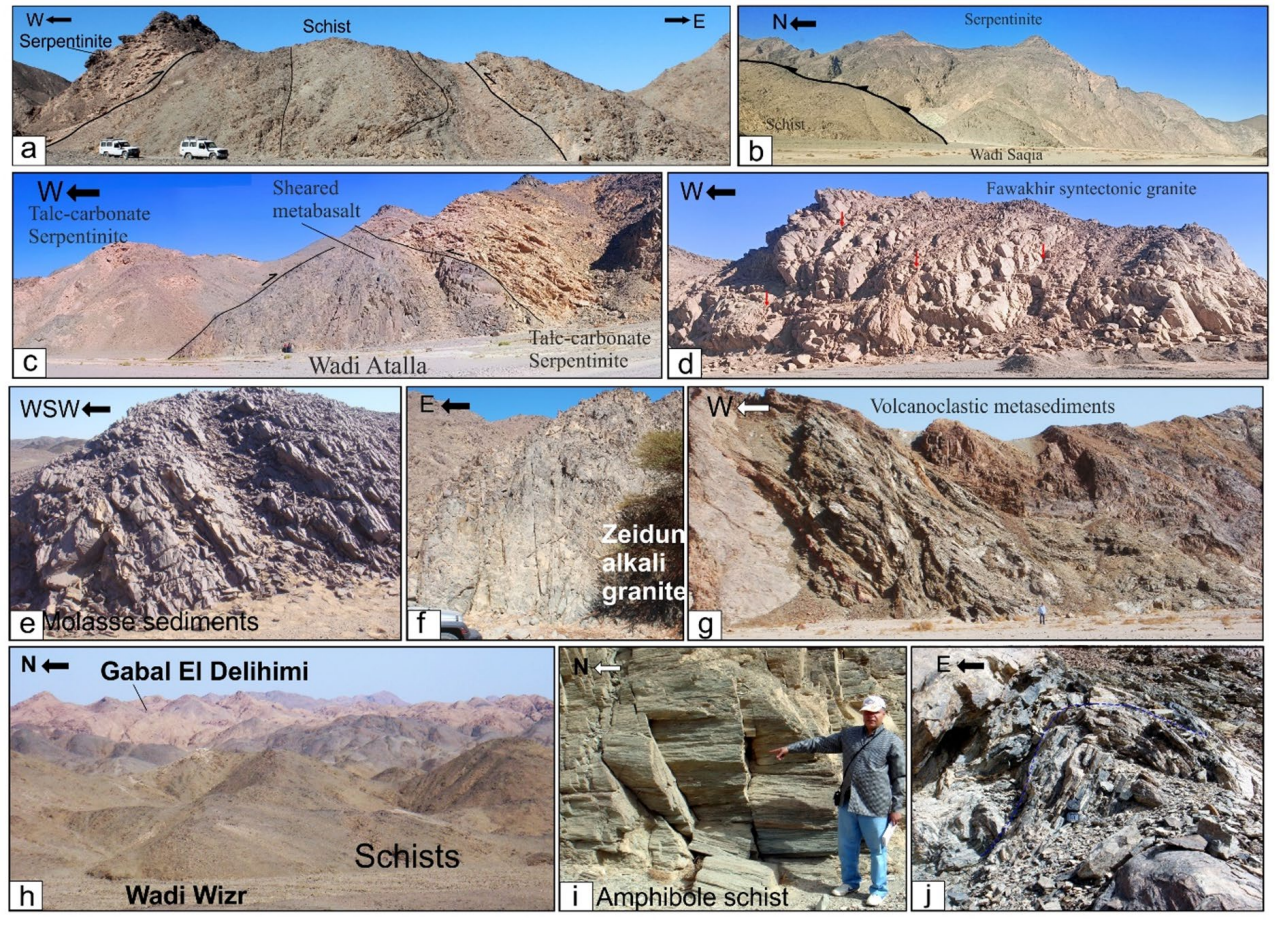
(**a**) N-S-striking thrusts between serpentinite and volcanoclastic metasediment, Safaga-Shalul shear zone, Wadi Homeiriyia (**b**) N-S-striking thrust between schist and Sagia serpentinite mass that extend parallel to the strike of the, Safaga-Shalul shear zone, Wadi El Saqia, (**c**) N- trending lenses of schistose metabasalts within the serpentinites of Wadi Um Esh El Zarga, Safaga-Shalul shear zone, (**d**) N-S-striking bands of mylonitic granite in the Fawakhir synorogenic tonalite, (**e**) Molasse sediment in Wadi Zeidun striking parallel to the Wadi Kareim-Umm Bisilla shear zone, (**f**) Nework of N-S trending fracture in Zeidun Alkai granite, (**g**) The N-S-striking subvertical S3 foliation in volcanoclastic metasediments at the entrance of Wadi Um Gheig, (**h**) Foliation in volcanoclastic metasediments striking N-S parallel to the long axis of Gabal El Delihimi granite, (**i**) L_3_ stretching subhorizontal lineation in amphibole schist along Um Gheig-Nugrus shear zone, Wadi Um Gheig. The person in the figure is the first author, (**j**) Symmetrical F3 anticline in volcanoclastic metasediments at the entrance of Wadi Um Luseifa.

In the northern region, serpentinites at Gabal Rubshi and Wadi El Saqia are oriented N-S (Fig. 6a), parallel to the primary plane of the SSSZ. The imbricate thrust sheets are made up of serpentinite and volcanoclastic metasediments (Fig. 6a). They also have an N-S strike, moving westward and creating noticeable structures that run along the SSSZ (Fig. 6b). Within the Abu Marawat area, the deformation is classified into high-strain zones that display simple shear combined with flattening strain and surrounding regions of considerably lower strain that are characterized by pure shear deformation^6^.

Rocks in the northern region exhibit significant shearing along the SSSZ, resulting in mylonites and ultramylonites with a distinct foliation (S3m). The SSSZ limits the steeper lineation plunges to the subordinate shear zone. Fabric elements’ NW-SE orientation shifts to N-S and NNW-SSE near the SSSZ^6^. The Abu Gaharish monzogranite, formed late in the orogenic phase, has auriferous quartz veins and alteration zones, indicating that its eastern boundary was sheared flexurally. Gold mineralization associated with quartz veins and alteration zones supports the presence of shear zones.

The core of SSSZ includes the Fawakhir ophiolitic rocks, granitoids, parts of the Meatiq and Um Had domes, and the Atalla shear zone, which is a NW–SE strike-slip zone exhibiting sinistral shearing. Within this area, a lens-shaped mass of sheared metabasalt is embedded in talc carbonate serpentinite, forming an N-S fabric that overlays the NNW fabric of the shear zone (Fig. 6c). A series of N–S-oriented thrust sheets made of metavolcanic and metagabbro are thrust westward over serpentinite, forming an imbricate thrust system intruded by the Fawakhir granitoids^6^. The Fawakhir grey tonalitic phase, dated to 638 Ma^108^, is fine- to medium-grained, with variable S3 foliation levels, producing mylonitic tonalite (Fig. 6d).

Shalul gneiss, volcanoclastic metasediments, and Hammamat molasse sediments predominantly define the southern domain. The molasse basins are thought to have formed in a N-S extensional setting with a long period of left-lateral transtension and NNE-trending synformal folds during ENE–WSW compression. The molasse sediments exhibit linear features, minimal reverse movement, and lengths exceeding 30 km, leading to the juxtaposition of hanging-wall metavolcanics adjacent to the footwall molasse sediments.

The Shalul monzogranite gneisses are characterized by an open domal structure oriented in a north-northwest to south-southeast direction. An N-S-striking thrust fault delineates the interface between the volcanoclastic metasediments and the Shalul gneisses, which exhibit a westward movement direction^6^.The volcanoclastic metasediments are marked by stretching lineations that trend north-northwest to south-southeast, while the foliation within these metasediments, which initially strikes northwest, transitions to an N-S orientation within the SSSZ^6^. Additionally, the NE-SW shear fabric (S4) in the Qena-Safaga shear zone affects the Barud granite north of the study area. Around Wadi El Bula, this shear zone comprises irregular, curved splays that affect the metagabbro, amphibolite, and hornblende schist.

#### Wadi Kareim-Umm Bisilla shear zone

**Fig. 7:**
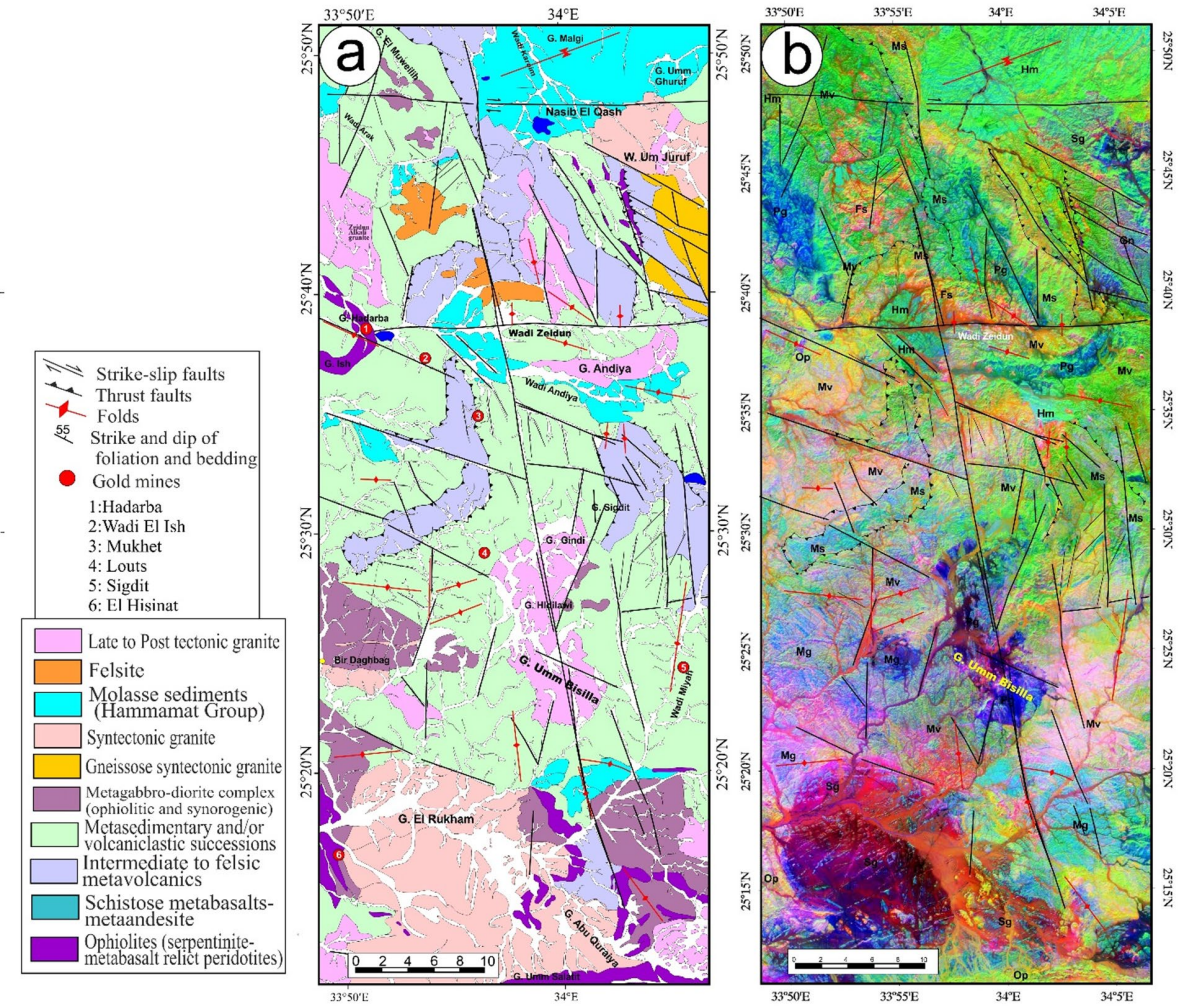
(**a**) Geological map of Wadi Kareim-Umm Bisilla shear zone (complied and modified from Hamimi et al.^109^; Hassan et al.^112^; El-Qassas et al.^128^; Conoco^129^; EGSMA^130^. (The figures were created by ArcGIS Desktop v 10.8. https://www.esri.com/en-us/arcgis/products/arcgis-desktop/overview, and SmartSketch v. 4.0 software; https://smartsketch.software.informer.com/4.0/). (**b**) PC415 in RGB of Landsat-8 showing the extension and major structures in the Wadi Kareim-Umm Bisilla shear zone. (The figure was created by ENVI v. 5.6.2. software; (https://www.l3harrisgeospatial.com/Software-Technology/ENVI).

Wadi Kareim-Umm Bisilla region (Table 2; Fig. 7a and b) comprises serpentinite, metagabbro, metavolcanics, and metasediments, as well as calc-alkaline foliated quartzdiorite to granodiorite (syntectonic granite), calc-alkaline granite^109^ and alkali-feldspar granite (post-tectonic granite). Moreover, ophiolite fragments composed of metaultramafics, metagabbros, and arc metavolcanics are embedded in a matrix of volcaniclastic metasediments, with thrust contacts interspersed throughout.

At Gabal Hadarba, serpentinite and related lithologies are found alongside metavolcanic formations, mainly composed of metamorphosed basic and intermediate metavolcanics (Fig. 7a and b). A notable metabasalt belt, which has been altered into amphibolite, extends from the northern part of Wadi Zeidun to G. Hadarba, following a NW-SE orientation^110^. The serpentinite and associated rocks are arranged into two intersecting belts that trend NW-SE and NE-SW, converging at G. Hadarba. The NW-SE belt includes light-gray talc-tremolite, actinolite rocks, situated above black serpentinite and beneath tuffaceous serpentinized metabasalt^110^.

The Umm Salatit district (Fig. 7a and b), in the southern Wadi Kareim-Umm Bisilla area, features diverse geological formations like ophiolitic mélange, metavolcanics, gabbro-diorite, tonalite, and monzogranite. It contains allochthonous blocks and clasts of serpentinite in the mélange, with carbonatized and silicified derivatives. These rocks are arranged in sheets, fragments, and blocks, thrust over island-arc metavolcanics in Gabal Umm Salatit^111^. Ultramafic rocks show linear zones of tectonized serpentinite with alterations along thrusts and shear zones, forming gold-mineralized listwaenite ridges (Fig. 7a and b).

The metasedimentary rocks mainly consist of interlayered metamudstones, metagraywackes, metaconglomerates, and schists. These are unconformably overlain by Hammamat molasse sediments in the Kareim and Andiya basins (Fig. 7a **and b**). Post-Hammamat features include Felsites, Natash Volcanics, and trachytic plugs, especially between Wadi Zeidun and Wadi El-Qash. Felsite intrusion has altered the molasse. A northwest-oriented microgranite dyke crosses the Arak basin. The Zeidun Alkali granite intrusion is N-S elongated and intrudes the volcaniclastic metasediments and metavolcanics of Wadi Arak. The Wadi Kareim basin is a significant east-west oriented syncline, surrounded by post-tectonic granitic formations, felsites, and bostonite dykes. Its molasse includes red and greenish-grey layers with conglomerates, wackes, siltstones, and sandstones. Normal faults trend NE-SW at their boundaries (33). Along the NE edge, shearing from sinistral-northwest-strike-slip faults is evident. Coarse conglomerates are at the top. Deformation includes initial syncline formation and regional NW-SE shearing linked to the Najd Fault System^33^.

The Wadi Kareim-Umm Bisilla region is characterized by the intrusion of various granitoids and mafic rocks, including diorite, olivine gabbro, granodiorite, monzogranite, and Alkali granite. This area is notable for significant ore deposits, including radioactive minerals such as uranothorite, thorite, zircon, fergusonite, xenotime, and chromite, as well as gold, all associated with hydrothermal alteration zones^112,113^.

**Fig. 8:**
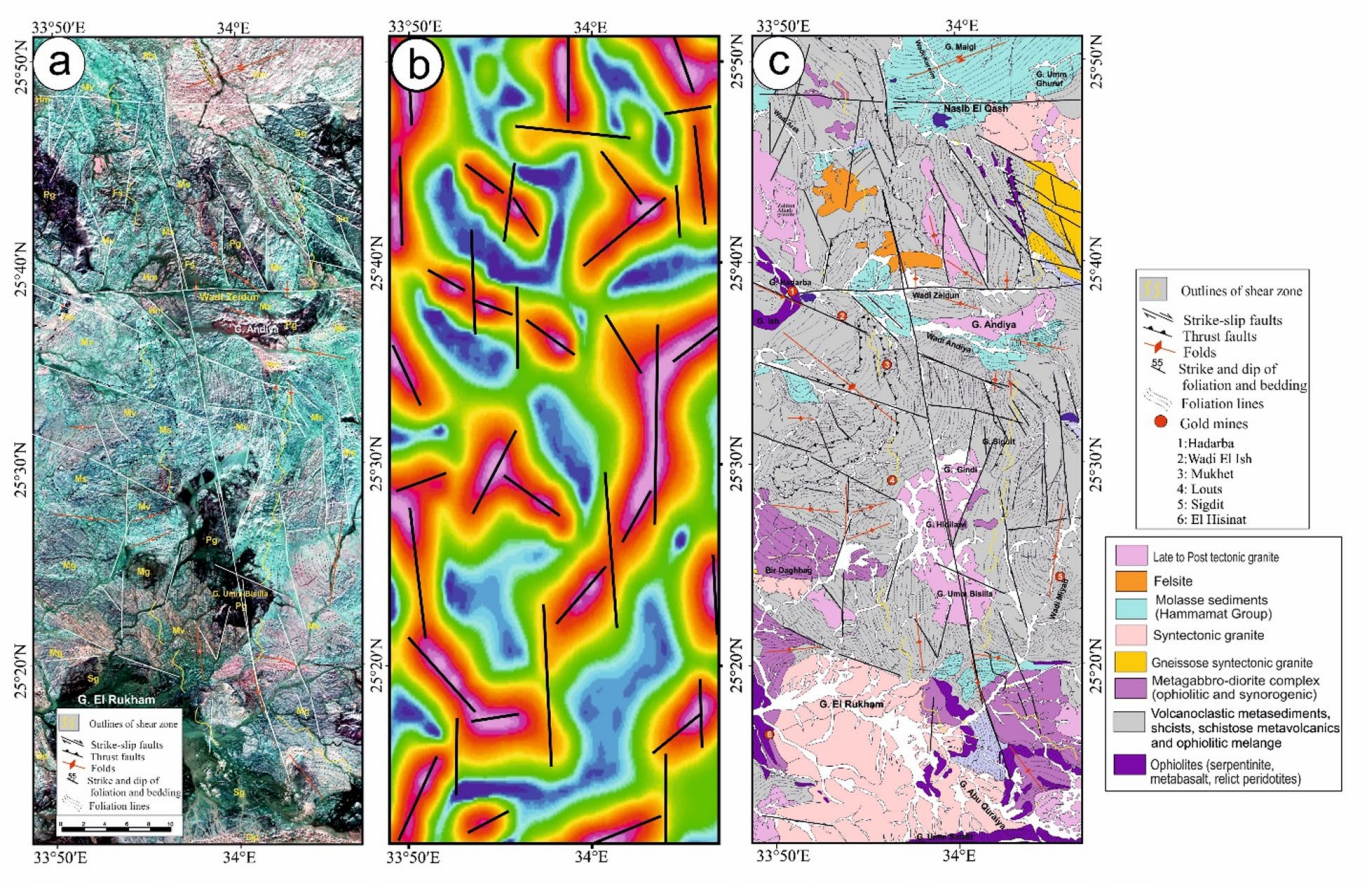
(**a**) Band ratio of 1/7, 3/5, and 2/5 for the Wadi Kareim-Umm Bisilla shear zone showing major structures and foliation lines. (The figure was created by ENVI v. 5.6.2. software; (https://www.l3harrisgeospatial.com/Software-Technology/ENVI), (**b**) The LTHG map of Wadi Kareim-Umm Bisilla shear zone, (The figure was created by Geosoft Oasis montaj v. 8.3.3. https://www.seequent.com/products-solutions/oasis-montaj/). (**c**) Structural map of Wadi Kareim-Umm Bisilla shear zone. (The figures were created by ArcGIS Desktop v 10.8. https://www.esri.com/en-us/arcgis/products/arcgis-desktop/overview, and SmartSketch v. 4.0 software; https://smartsketch.software.informer.com/4.0/).

The structural maps of the Wadi Kareim-Umm Bisilla region (Fig. 8a, b, **and c**) illustrate three distinct phases of foliation and folding. The LTHG map of this region (Fig. 8b) shows a dominant N-S trend with NW-SE, NNE-SSW, and NE-SW directions. The S1 foliation remains present in the F2 fold hinges and limbs, as well as in older imbricated thrust sheets located between serpentinite, volcanoclastic metasediments, and metavolcanics. These imbricated thrust sheets are observed at multiple scales, from the microscopic to the outcrop level. Notably, the dip angles of the thrust planes tend to be steeper in the down-dip direction, while the up-dip side exhibits gentler angles. Within the volcanoclastic metasediments, the F2 folds are mainly oriented northwest-southeast, as evidenced north of Bir Daghbag and Gabal Andiya. S2 represents an axial planar foliation associated with F2 folds, which significantly impacts the ophiolite mélange. These folds are characterized by both overturned and tightly plunging mesoscopic structures. The area features two prominent NW-trending folds. The Gabal Hadarba anticline consists of folded volcanoclastic metasediments and serpentinite, with its two limbs corresponding to the Gabal Hadarba and Gabal Ish serpentinites. The core of this structure consists of ophiolitic mélange and schists. To the south of the Gabal Hadarba anticline lies another major NW-trending anticline (Fig. 8a, b, **and c**), where older imbricated thrust sheets, volcanoclastic metasediments, and metavolcanics are folded around an NW-plunging axis. The hinge of this anticline is offset by a sinistral strike-slip fault striking NW-SE (Fig. 8a, b, **and c**). In the southwestern part of the mapped area, a significant NW-trending fold is present within the ophiolitic mélange of Gabal Umm Salatit. During this geological event, a layer of molasses-like sediments accumulated in basins connected to shear zones.

The N-S shear fabric marks the KBSZ boundaries, extending about 70 km from Gabal Umm Salatit to Wadi Karein. The zone shows increasing strain toward the center, with numerous mylonitic zones ranging from 10 to 200 m wide, some up to 10 km wide. These zones feature nearly vertical foliation and almost horizontal lineation. The S3 foliation reveals that the KBSZ consists of north-trending, sub-parallel, and anastomosing lineaments.

Furthermore, this shear zone is linked to several parallel shear zones and splay faults within its central core. The S3 foliation in schists and volcanoclastic metasediments and sheared metavolcanics, as well as fracture cleavage in the Zeidun molasse sediments (Fig. 6e), are consistent strikes from N-S to NNE-SSE and typically show subvertical or steep dips towards the NE or SW, with angles generally ranging from 55 to 75°. Additionally, the orientation of fabric elements transitions from NW-SE to N-S and NNW-SSE in the vicinity of the KBSZ.

A series of N-S trending thrusts has developed north of Wadi Zeidun (Fig. 8a, b, **and c**). These imbricate thrust sheets, composed of metavolcanics and volcanoclastic metasediments, are oriented N-S and move westward, forming prominent structures that extend along the KBSZ in a north-south direction. The F3 folds mainly trend N-S and NNE-SSW, with a predominant plunge toward the north (Fig. 8a, b, **and c**). A network of dextral strike-slip shear zones in the KBSZ indicates active thrust-dominated transpression. The presence of many positive flower structures suggests that thrusting in the KBSZ is not merely a simple thrust system but rather a sign of a transpressional regime^109^. Kinematic indicators and structural features, such as folding within shear zones, S-C foliation, steeply dipping frontal thrust ramps, deflected bands, and asymmetric porphyroclasts and mineral fish observed at the microscopic level, all point to strike-slip movement or transcurrent shearing^109^. These movements can cause shear zone-related folding and, in some cases, restraining bends in specific exposures^109^.

A notable feature of the KBSZ is the intrusion of the Zeidun and Um Bisilla alkali granite plutons, which have their long axes aligned parallel to the primary strike of the KBSZ. The Zeidun alkali granite is characterized by a network of N-S-trending fractures (Fig. 6f) running parallel to the strike of the KBSZ.

At Gabal Umm Salatit, the southern part of the KBSZ is divided by large dextral strike-slip shear zones of the Mubarak-Baramiya shear belt, which run NE–SW. The foliations, called S4, strike ENE–WSW with a dip of 35°–55° to the southwest, and are aligned with F4 folds. High-strain zones along thrust planes are typically foliated NE–SW with dips of 45–65°, showing widespread cleavages and pencil fabrics from intersecting fracture sets. Dextral transpression is characterized by crenulations, mineral lineation, rods, boudinage, and kink bands, forming large shear zones and thrusting ultramafics over metavolcanics. The KBSZ also shows an overprint of the S4 shear fabric associated with the Wadi Ziedun dextral shear zone.

Faults and lineament features in the area are aligned in consistent NNE–SSE, NW–SE, and NE–SW directions. A prominent strike-slip fault is identified at the centre of the KBSZ. This sinistral strike-slip fault extends over 80 km, displacing all rock units, and it appears to have been reactivated along the core of the older Wadi Kareim-Umm Bisilla dextral strike-slip shear zone.

#### Um Gheig-Nugrus shear zone (GNSZ)

**Fig. 9:**
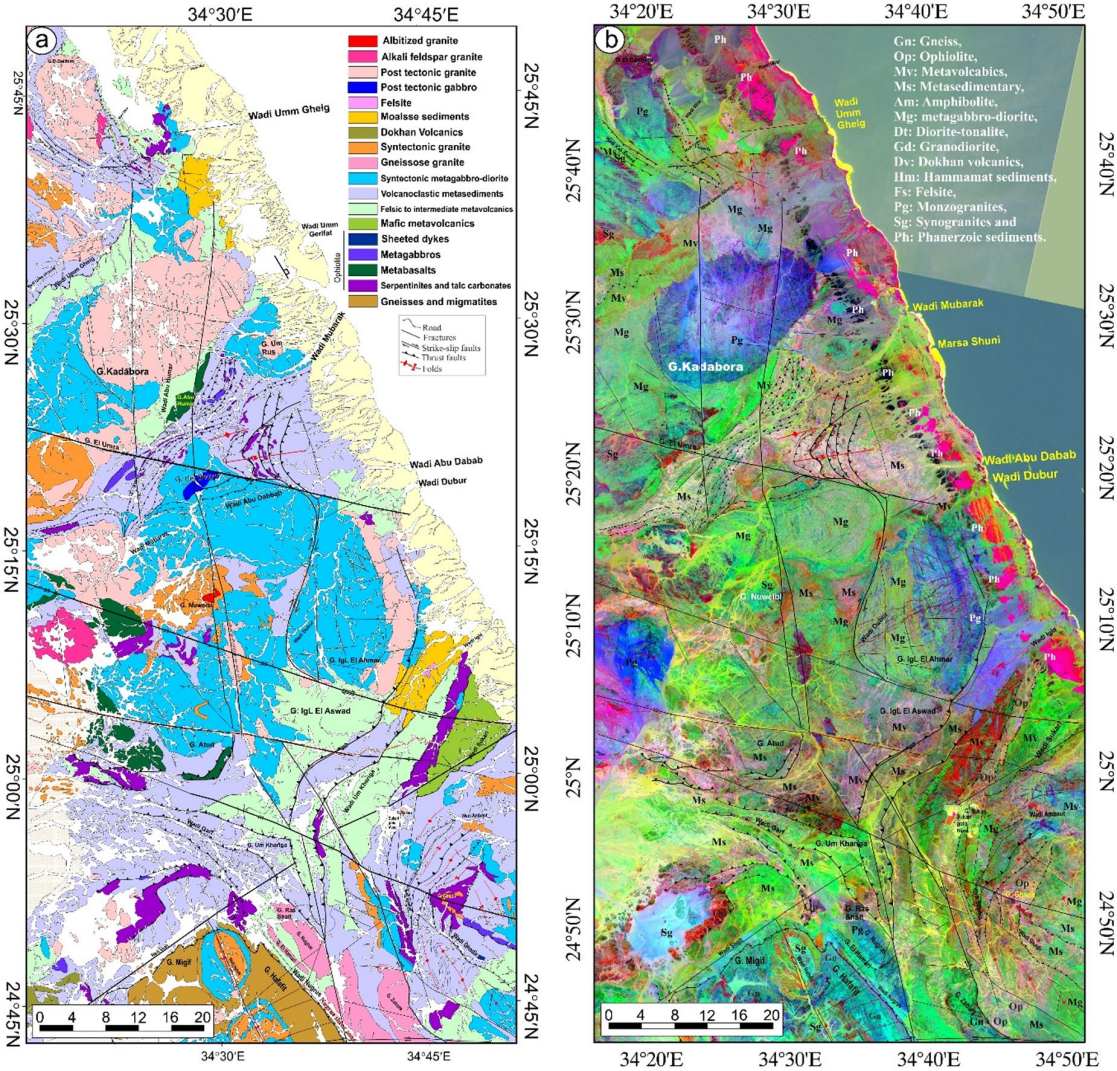
(**a**) Geological map of Um Gheig-Nugrus shear zone (GNSZ) (complied and modified after Abd El-Wahed et al.^58,59^; Abd El-Wahed and Kamh^60^; Hamdy et al.^117^; El-Sayed^118^; Conoco^129^; EGSMA^130^, (The figure was created by ArcGIS Desktop v 10.8. https://www.esri.com/en-us/arcgis/products/arcgis-desktop/overview, and SmartSketch v. 4.0 software; https://smartsketch.software.informer.com/4.0/). (**b**) MNF3, MNF4, and MNF2 in RGB, showing lithological variation and major structures in the Um Gheig-Nugrus shear zone area. (The figure was created by ENVI v. 5.6.2. software; (https://www.l3harrisgeospatial.com/Software-Technology/ENVI)

The Wadi Um Gheig area (Table 2) hosts various geological units, including ophiolitic associations, metavolcanic suites, granitoids, and Hammamat sediments (Fig. 9a, b). The ophiolitic suite includes serpentinites, metagabbros, hornblende schist, and volcaniclastic metasediments^58,113,114^. Serpentinite and talc-carbonate lenses occur within volcaniclastic metasediments and hornblende schist, forming a tectonic mélange. The metagabbros range from fine- to medium-grained and retain original igneous layering. Hornblende schist forms NNW-SSE belts thrust over volcaniclastic metasediments. The area also includes meta-andesites, spilitic meta-basalts, and felsic metavolcanics, which are foliated and increasingly transformed into schists^58,114^.

Non-foliated metavolcanics exhibit a distinct boundary with foliated metavolcanics. The foliated metavolcanics are juxtaposed against Hammamat sediments and are bordered by ophiolitic blocks. Late to post-tectonic granites include Umm Shaddad alkali feldspar granites and the El Deilihimmi granodiorite. Hammamat molasses sediments form isolated hills composed of coarse- to fine-grained conglomerates interspersed with greywacke.

The region west of the Kadabora pluton comprises disordered blocks and fragments of serpentinite, metagabbro, and amphibolite, all set within a metasedimentary mélange matrix^58,115^. The arc granitoids comprise various igneous rocks, such as quartz diorites, tonalites, and granodiorites, which are significant formations characterized by numerous joints and fractures^58,115^.

The Wadi Mubarak region features diverse rocks, including metavolcanics, metatuffs, volcaniclastics, and magmatic formations. Metavolcanics are NE-SW oriented, while metatuffs are intermediate to acidic with various fragments (Fig. 9a **and b**). Interbedded volcaniclastic metasediments form a tectonic mélange^31,60,81,116^. The belt shows thrust sheets, including ophiolitic metagabbros, metabasalts, pillow lavas, and serpentinites, as lenticular masses and small sheets, some of which are discordant to bedding and foliation^60,116^. The area also hosts syn-tectonic intrusions, including the Gebel El-Umra and Gebel Abu Karanish granites, and the Mubarak-Dubr metagabbro-diorite complex. The El-Umra granite is mainly quartz diorite to granodiorite, emplaced 690–645 Ma^31^. The Mubarak-Dubur complex includes hornblende metagabbros, diorites, uralitized gabbros, metadiabases, and amphibolites, subdivided into metadiorite and metagabbro associations, and olivine and hornblende metagabbros^81,117^. Geochemical data indicate that the complex is an island arc with calc-alkalic and minor tholeiitic features^118^. It is intruded and faulted against metasedimentary rocks and granites, with serpentinite masses included.

Post-tectonic intrusives encompass gabbros, such as those found at Gabal Um Battat (Fig. 9a **and b**) and Gabal Um Rus, as well as post-tectonic granites, including Igl El-Ahmar monzogranite, Kadabora monzogranites, Nuweibi apogranites, and Dubur perthitic leucogranites^119^. Nuweibi apogranites, the most recent phase of granite formation, are characterized by rare-metal mineralization and are associated with Mesozoic magmatism^120^.

The basement succession in the Gabal Atud area is divided into three main rock groups: (1) ophiolitic mélange units, (2) island-arc assemblages, and (3) post-collisional mafic intrusions (Fig. 9a **and b**). Notably, the metagabbro-diorite complex and the fresh gabbros of G. Atud are rich in gold, hosting the Atud gold mine. Evidence of intrusive contacts between gabbroic rocks and nearby rock units is present, with metagabbro-diorite rocks showing multiple pulses that penetrate serpentinites, metatuffs, and metaconglomerate-metagreywack^121^.

The Um Khariga region (Fig. 9a **and b**) features various geological formations, including metavolcanics, volcaniclastic metasediments, and serpentinites. Near Wadi Um Khariga, metavolcanic rocks mainly consist of basaltic and dacitic-rhyolitic flows and volcaniclastics. Near Wadi El Sukari, a sequence of coarse metatuffs, lapilli metatuffs, and metabasalt agglomerates exists. The metapyroclastic materials include fine- and coarse-grained metatuffs, lapilli metatuffs, agglomerates, and lesser metadacite and metarhyodacite flows^81^. Serpentinites are dissected, elongated masses with sheared and foliated edges. Metavolcanics are intruded by metagabbro-diorite, dolerites, and alkali feldspar granite from Gebel El Sukari^81^. Over the metavolcanics lie molasse-type sediments in Wadi Igla. The Sukari gold mine is the largest in the Central Eastern Desert.

The Wadi Ghadir region is characterized by the Ghadir ophiolite, which comprises an ophiolitic mélange of fragments from ophiolitic sheets, greywacke, siltstone, mudstone, and granitic rocks^59^. The serpentinized peridotites in the area can be categorized into two distinct types: massive peridotites and foliated peridotites, the latter exhibiting significant shearing and altered talc-carbonate rocks. The upper section of the cumulate sequence is composed of gabbroic rocks situated beneath the sheeted dyke complex (Fig. 9a **and b**). Abd El-Rahman^70^ classified the plutonic rocks of the Wadi Ghadir ophiolite into three categories: layered, coarse-grained massive, and hypabyssal gabbros. The basal unit is the layered gabbro, which transitions upward into the massive coarse-grained gabbro, culminating in the hypabyssal gabbro at the top^59^. Zabara granite gneisses include granite gneiss with hornblende gneiss, as well as minor migmatites, cataclastics, mylonites, and schists^122^.

The Nugrus region is characterized by various geological formations, including meta-ultramafics, island-arc assemblages, hornblende-biotite gneiss, and granitoid rocks (Fig. 9a **and b**). The granitoids in this area are classified as syn-tectonic and late to post-tectonic, with deformed granitoids constituting the Hafafit domes. These rocks are predominantly found at two significant outcrops: mylonitized biotite granite at Gabal Nugrus and mylonitized muscovite. The granitic rocks exhibit a notable mylonitic texture and are influenced by a principal N-S trending strike-slip fault and several minor normal faults^123^.

The Nugrus region is located in the southeastern part of the Hafafit gneissic complex (Fig. 9a and b), mainly composed of mafic gneisses with tonalitic gneisses at its center^124^. It features gneiss domes and the Nugrus shear zone, a notable ductile shear zone separating the lower gneisses of the Hafafit complex from the overlying lower-grade ophiolitic serpentinites, metagabbros, and metavolcanics and metasediments related to the Ghadir ophiolite^125^. Geochronological data from the tonalitic intrusions within the Hafafit gneissic complex, obtained by U-Pb and Pb-Pb zircon dating, indicate ages of around 680 Ma (Lundmark et al., 2012). Rb-Sr dating provides an age of 609 ± 17 Ma^126^, likely reflecting shearing activity along the Nugrus shear zone^124^. This shearing event occurred shortly before the Ar- Ar hornblende cooling ages of 584–586 Ma recorded in the Hafafit gneissic complex^127^. Additionally, the Nugrus shear zone hosts syn-tectonic granites, such as biotite orthogneiss and muscovite granite, representing the latest phase of granitic intrusions in this region^34^.

**Fig. 10:**
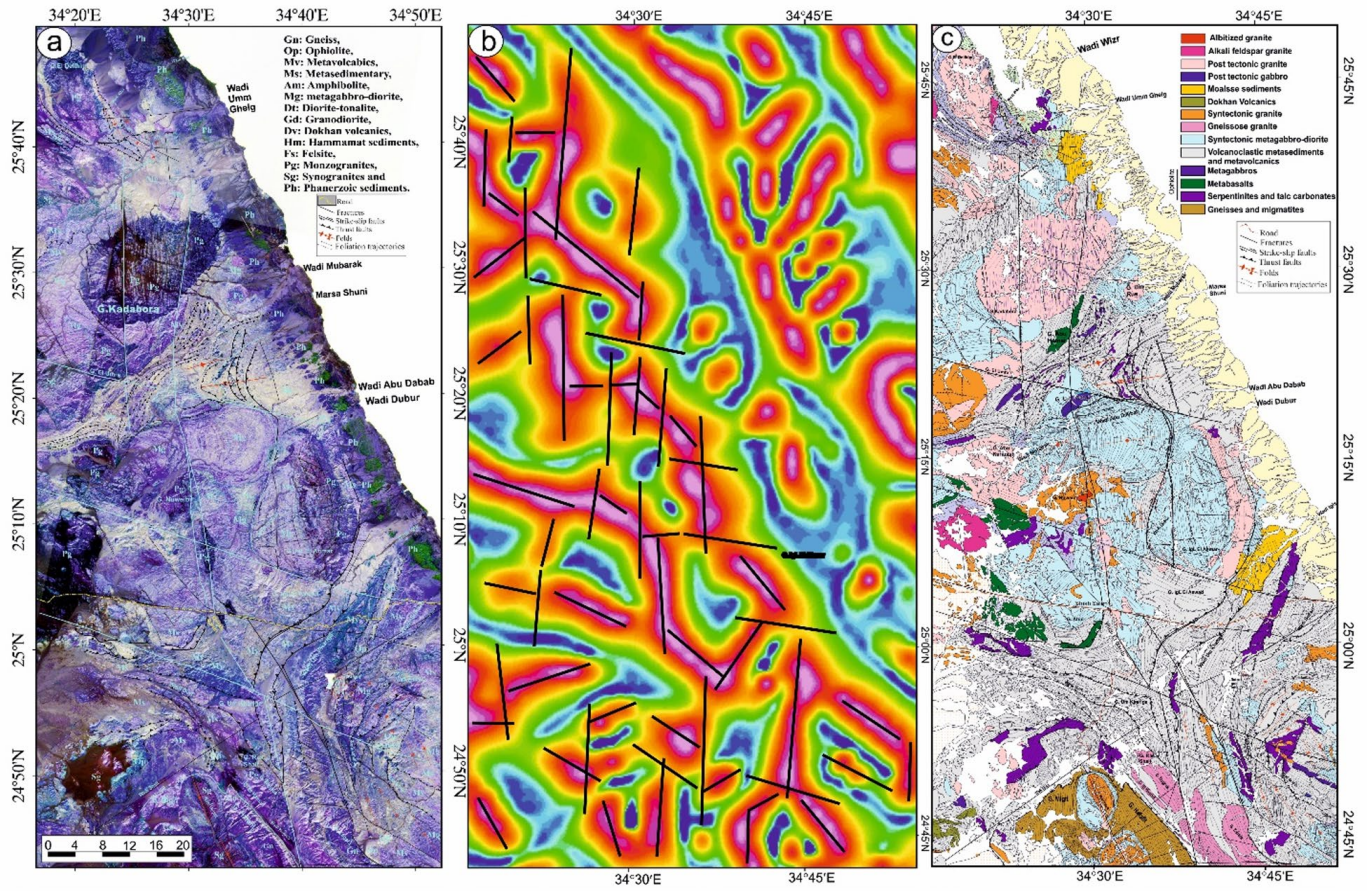
(**a**) Band ratio of 6/3, 7/2, and 7/1 for the Um Gheig-Nugrus shear zone showing major structures and foliation lines. The figure was created by ENVI v. 5.6.2. software; (https://www.l3harrisgeospatial.com/Software-Technology/ENVI), (**b**) The LTHG map of Um Gheig-Nugrus shear zone. The figure was created by Geosoft Oasis montaj v. 8.3.3. (https://www.seequent.com/products-solutions/oasis-montaj/). (**c**) Structural map of Um Gheig-Nugrus shear zone. The figures were created using ArcGIS Desktop v10.8. https://www.esri.com/en-us/arcgis/products/arcgis-desktop/overview, and SmartSketch v. 4.0 software; https://smartsketch.software.informer.com/4.0/).

Structurally, the regions of Um Gheig, Hafafit, Ghadir, and Garf exhibit a predominance of NW-SE-oriented fabric. At the same time, the areas of Mubarak, Um Khariga, and Sukari are characterized by a NE-SW-oriented fabric (Fig. 10a-c). Figure 10b indicates that the N-S, NW-SE, NNE-SSW, E-W, and WNW-ESE are the main structural trends identified on the LTHG map of the GNSZ. The GNSZ comprises a network of interconnecting ductile shear zones that trend N-S, NNW-SSE, and NNE-SSW. The oldest foliation, designated as S1, trends E-W and has been modified by various deformation events, resulting in the formation of NW-SE (S2), N-S (S3), and NE-SW to ENE-WSW (S4) foliations, along with associated folds. The NW-SE-oriented fabric (S2) is particularly prominent in the Um Gheig, Hafafit, Ghadir, and Garf regions. In contrast, the N-S-oriented fabric is prevalent in Wadi Wizr, located to the north and south of Gabal Kadabora, south of Gabal Um Battat, and in the area between Gabal Ras Shait and Wadi Sabahia, extending northward to Wadi El Barda and Sheikh Salim (Fig. 10a, b, **and c**). The regions of Wadi Mubarak, Um Khariga, and Sukari are dominated by NE-SW-oriented fabric, corresponding to S4 foliation.

In the Um Gheig region, a sequence of NW-trending anticlines and synclines (Fig. 10a, b, **and c)** is relatively open, characterized by a general asymmetry with steep forelimbs and back limbs. These geological folds exhibit high amplitudes and are spaced approximately 2 to 10 km apart, with wavelengths reaching up to 2 km. The primary deformation event, designated D2, has produced D2 structures, including significant macroscopic folds (F2), an associated penetrative axial-plane schistosity (S2), and a mineral lineation (L2). The S2 foliations predominantly display a preferred orientation with a northwest strike; however, irregular continuous foliations striking N-S and WNW-ESE are also notable, influenced by subsequent deformation events. The N-S-striking subvertical S3 foliation (Fig. 6g **and h**), L3 stretching subhorizontal lineation (Fig. 6i), and F3 folding (Fig. 6j) are distinctly visible in the volcanoclastic metasediments and sheared metavolcanics located along Wadi Umm Luseifa and at the entrance of Wadi Um Gheig (Fig. 10a, b, **and c**). Additionally, a series of prominent F3 folds has developed within the volcanoclastic metasediments along the western bank of Wadi Wizr.

In the Wadi Mubarak region, curvilinear structural patterns dominate, especially near the metagabbro-diorite interface. The area’s tectonics feature spaced cleavage (S4) trending NNE-SSW to NE-SW with steep dips ESE, SE, and occasionally NW (Fig. 10a-c). High-strain rocks mainly include fine-grained phyllonites and mylonite schists with steep foliation^31,60,81^. To the northwest, Gebel El-Umra syn-tectonic granitic gneiss shows significant deformation, shearing, and mylonitization. The regional S4 cleavage is subjected to moderate to strong folding around gently plunging axes NE or SW, producing decimeter- to meter-scale elliptical folds that range from open to tight, asymmetrically overturned, and recumbent^60,81^.

**Fig. 11:**
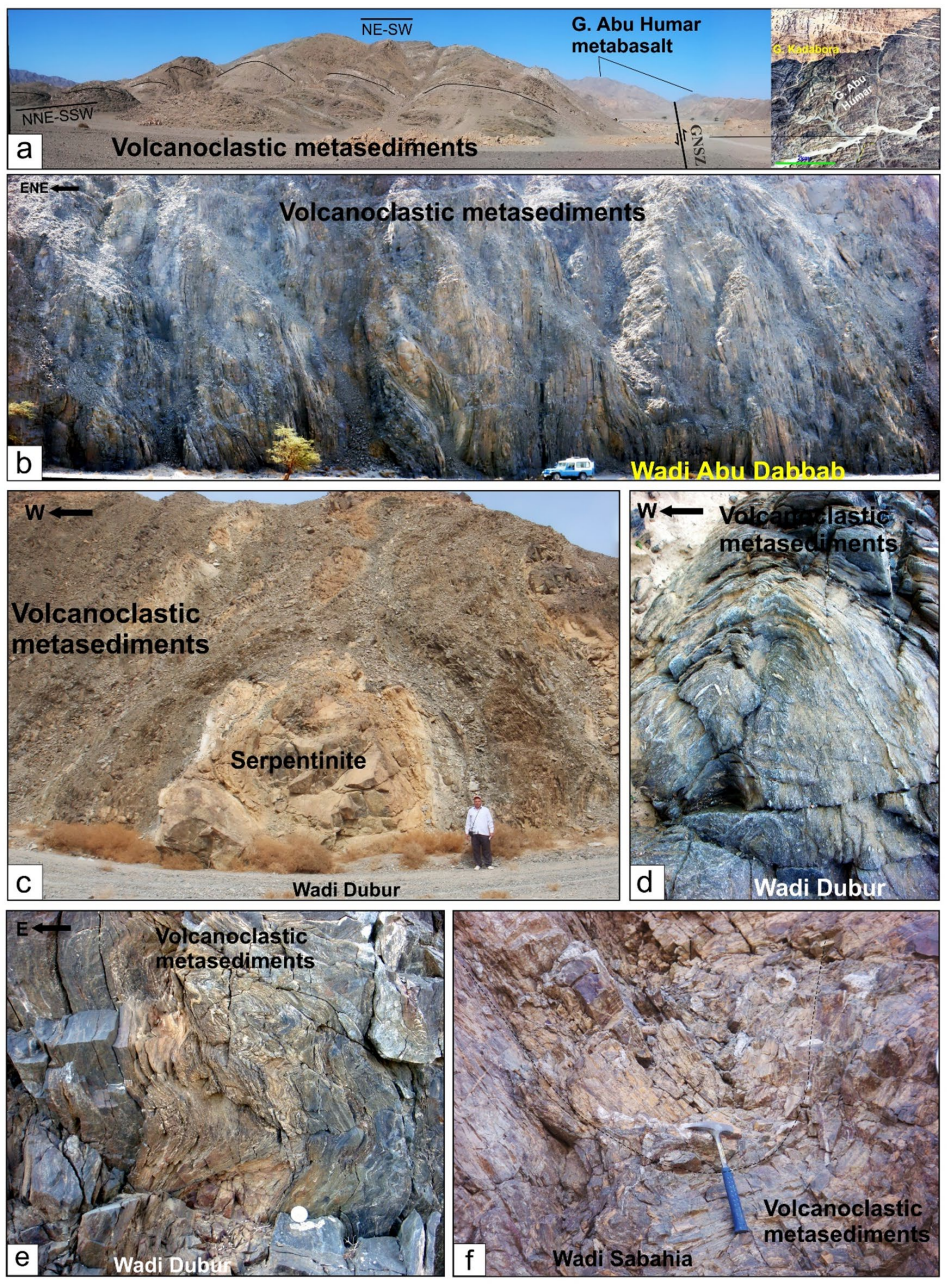
(**a**) Change in the fabric direction from NE-SW orientation to NNE-SSW and finally to a N-S direction along Wadi Mubarak, (**b**) N-S-striking subvertical foliation from the Um Gheig-Nugrus shear zone along Wadi Abu Dabbab, (**c**) Serpentinite mass within volcaniclastic metasediments characterized by N-S S3fabric within the Um Gheig-Nugrus shear zone along Wadi Dubur. The figure belongs to the first author, (**d**) and (**e**) F3 folds in volcaniclastic metasediments within the Um Gheig-Nugrus shear zone along Wadi Dubur, (**f**) NNW-plunging fold in volcaniclastic metasediments of Wadi Sabahia.

In the Wadi Mubarak region, the shear belt displays prominent kinematic features, including an S-shaped configuration in its central section, asymmetric porphyroclasts of quartz and feldspar, and crenulation cleavages. These characteristics suggest a dextral sense of shearing along NE-trending ductile strike-slip shear zones^31,60,81^. A notable change in the fabric’s direction is evident, moving from a NE-SW orientation to NNE-SSW (Fig. 10a, b, **and c**) and finally to a N-S alignment (Fig. 11a). This rotation of foliation is found north of Gabal Abu Karanish and south of Wadi Abu Dabbab (Fig. 11b), in the area between Gabal Um Rus and Gabal Um Sanyiat. The bending and rotation of foliation from NE-SW to NNE-SSW and N-S suggest that the NE-oriented fabric has been locally influenced by NNE-SSW-trending shearing (Fig. 11b).

The widest part of the GNSZ is found near Wadi Dubr, especially between Gabal Igl El Ahmar and Gabal Nuwebi (Fig. 10a, b, **and c**), where the N-S S3fabric (Fig. 11c) and the F3 folds (Fig. 11d **and e**) that go from N-S to southeast are mostly intact. In the metagabbro-diorite and volcanoclastic metasediments between Wadi Dubr and Wadi Abu Dabbab, the S3 fabric is overprinted by NE-trending dextral oblique shear zones, resulting in an ENE-WSW-trending fabric (S4) and significant folds (F4). In Wadi Dubur, the most prominent N-S-trending feature is the Dubur perthitic leucogranite intrusion, which parallels the primary core of the GNSZ (Fig. 10a-c).

The N-S-oriented fabric (S3) of the GNSZ extends from Shikh Salim through Wadi El Barda to the deflection at Wadi Nugrus, where it influences the NW-trending fabric and thrusts of Wadi Garf, Wadi Um Khariga, Sukari, and Wadi Ghadir (Fig. 10a, b, and c). Between Gabal Ras Shait and Gabal Ghadir, a highly mylonitized N-S trending fabric occurs, along with east- and west-dipping thrusts and north- to NNW-plunging folds (Fig. 11f), particularly evident along Wadi Sabahia and Gabal Zabara gneiss-granite. The rocks within this intensely mylonitized N-S trending zone consist of highly tectonized basic metavolcanics, serpentinites, talc-carbonate, and metagabbro-diorite, all set within a matrix of volcaniclastic metasediments and schists. The mineral stretching lineations display variable plunges, whereas the mylonitic foliation, which trends NNW in the volcaniclastic metasediments, curves toward a north- and northeast-oriented orientation. The thrusts exhibit a characteristic propagation sequence (Fig. 10a-c), forming pop-up and flower-like structures. This intensely mylonitic zone, oriented north-south, is surrounded by various fault sets, including reverse thrusts and vertical strike-slip shear zones (Fig. 10a-c). These thrusts define the tectonic boundaries between ophiolitic slabs and the underlying metavolcanics and metagabbro-diorite. Highly sheared talc schists, talc carbonates, and listwaenite are often associated with these thrusts and strike-slip shear zones. Slickenlines are found along shear planes with a 10-15° plunge toward N20°W, while fiber lineations show a steep plunge of 52° toward the ESE. The regional foliation near Gabal Ghadir strikes NNE-SSW but bends into an NE-SW orientation and further into an ENE-WSW direction (S4) along Wadi Ambaut, which has undergone folding due to F4. The N-S-trending fabric affects the northern portions of the gneissic granites at Gabal Nugrus and Gabal Zabara, but it is not recorded in the gneisses of Gabal Hafafit.

#### Barramiya-Mueilha shear zone (BMSZ)

**Fig. 12:**
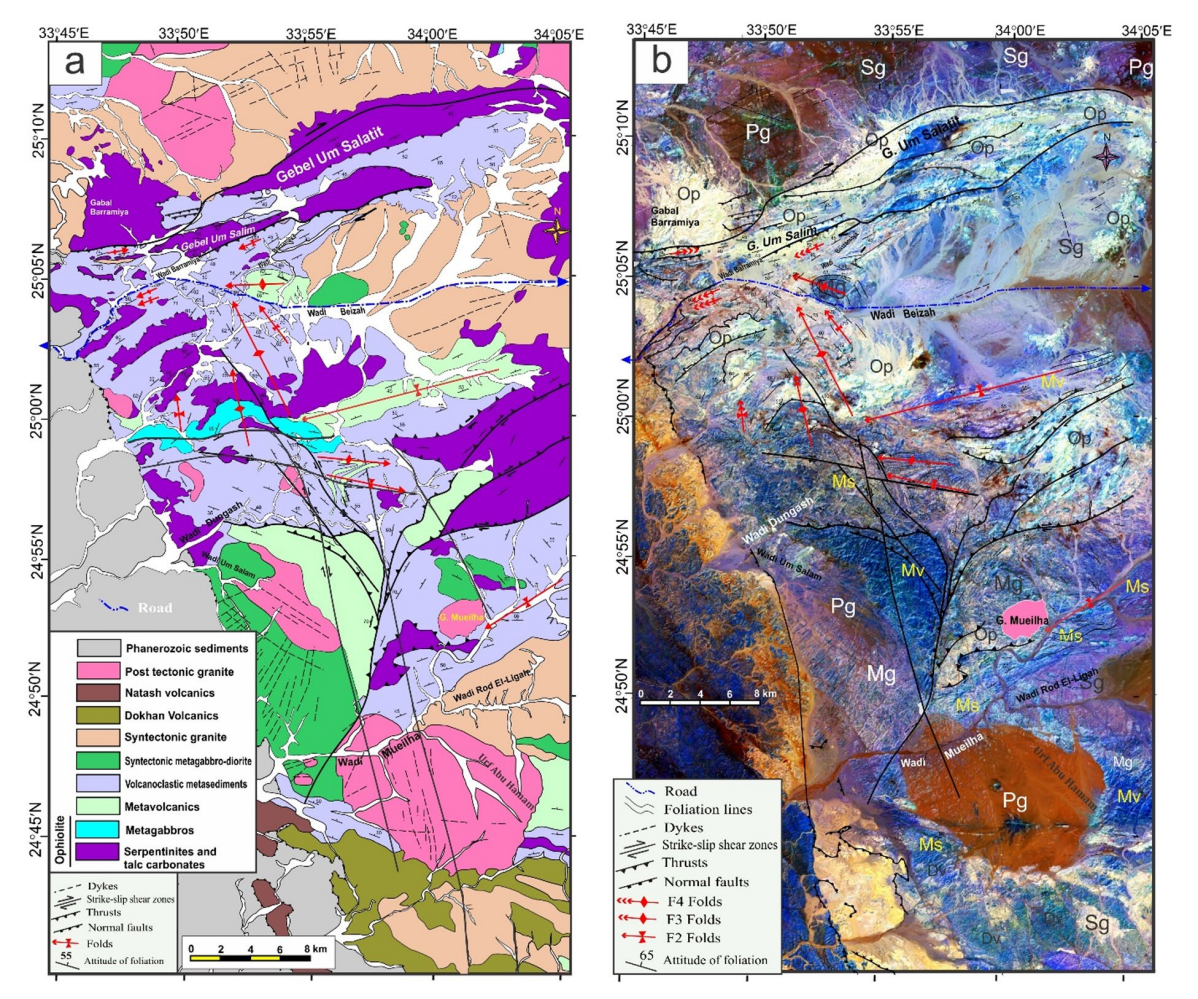
(**a**) Geological map of Barramiya-Mueilha shear zone (BMSZ) (compiled and modified after Abd El-Wahed^81^; Conoco^129^; EGSMA^130,138^; Zoheir et al.^131^). The figures were created using ArcGIS Desktop v10.8. https://www.esri.com/en-us/arcgis/products/arcgis-desktop/overview, and SmartSketch v. 4.0 software; https://smartsketch.software.informer.com/4.0/). (**b**) Band ratios of 4/6, 1/7, and 2/5 for the Barramiya-Mueilha area show lithological variation and major structures. (The figure was created by ENVI v. 5.6.2. software; (https://www.l3harrisgeospatial.com/Software-Technology/ENVI)

Ophiolites are mainly fragmented and define the Neoproterozoic rocks around the Barramiya-Mueilha shear zone (Table 2). These ophiolites consist of a tectonic mixture of serpentinite along with pelitic and calcareous schists (Fig. 12a **and b**). The metavolcanic rocks associated with the island arc include metabasalt, basaltic meta-andesite, and agglomerate. Near Wadi Beizah, weakly deformed metagabbro-diorite complexes cover large areas. The late or post-orogenic intrusions are mainly composed of alkali-feldspar granites and granite porphyries, and, to a lesser extent, albitite, as at G. Mueilha. In the western and northern parts of the study area, late or post-tectonic granites intersect the ophiolitic mélange, arc metavolcanics, and metagabbro-diorite formations^89,131^. Additionally, rhyodacite-rhyolite rocks and their subvolcanic counterparts are exposed in limited areas in the far southern part of the map area.

**Fig. 13:**
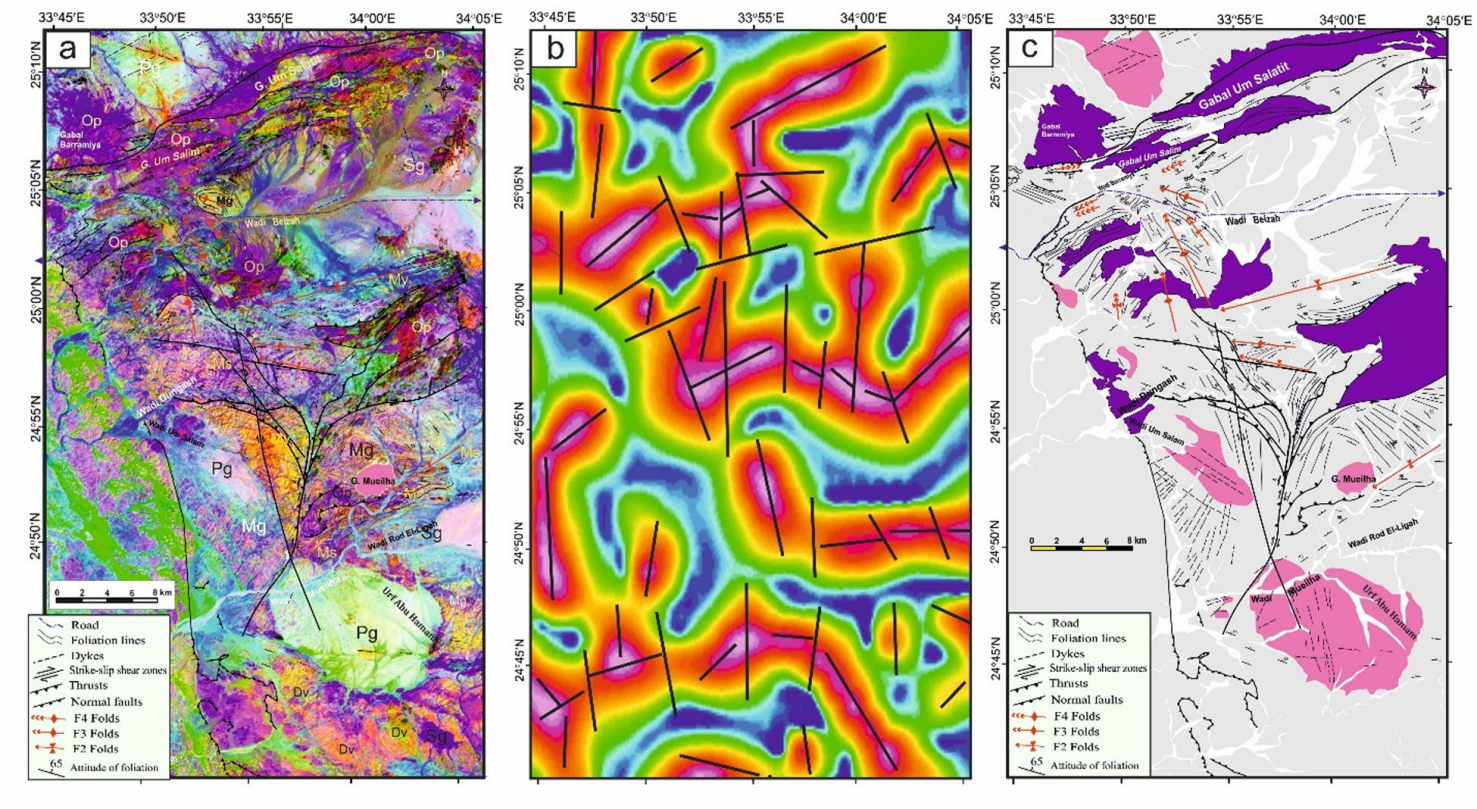
(**a**) MNF2, MNF3, MNF7 in RGB showing the extent and major structures along the Barramiya-Mueilha shear zone (BMSZ). The figure was created by ENVI v. 5.6.2. software; (https://www.l3harrisgeospatial.com/Software-Technology/ENVI), (**b**) The LTHG map of BMSZ, (c) Structural map of Barramiya-Mueilha shear zone. (The figures were created by ArcGIS Desktop v 10.8. https://www.esri.com/en-us/arcgis/products/arcgis-desktop/overview, and SmartSketch v. 4.0 software; https://smartsketch.software.informer.com/4.0/).

The Barramiya-Mueilha shear zone features a complex network of interconnected ductile shear zones that extend approximately 40 km from Wadi Mueilha in the south to Wadi Beizah in the north (Fig 13a, b, **and c**). The LTHG map of BMSZ (Fig. 13b) shows that the main trends in the area are NNW-SSE, NNE-SSW, WNW-ESE, N-S, and NW-SE. The structural features within this shear zone are interpreted as the result of four distinct phases of ductile deformation labeled D1 through D4. Moreover, the presence of extensional structures, such as fault and fracture zones, along with dike swarms cutting through the ductile formations in various orientations, indicates a later phase of brittle deformation (D4). D1 involves the stacking of significant ophiolitic nappes, with S1 foliation serving as a prime example. In the Barramiya and Dungash areas, serpentinite appears in steeply dipping belts from WNW to NE, positioned along thrust planes and aligned parallel to the widespread foliation in the ophiolitic mélange. The S1 foliation is characterized by ENE-striking shear features developed around the ophiolites, constrained by thrusts. The tectonic boundaries between the allochthonous serpentinite bodies and the metasedimentary mélange matrix are marked by schists, talc–magnesite formations, listvenite, and pockets of chromite. Additionally, steeply dipping mineral foliation (S2), oriented NW-SE to WNW-ESE, is most prominently observed in schistose rocks and in sheared metagabbro, meta-agglomerate, and metatuffs.

**Fig. 14:**
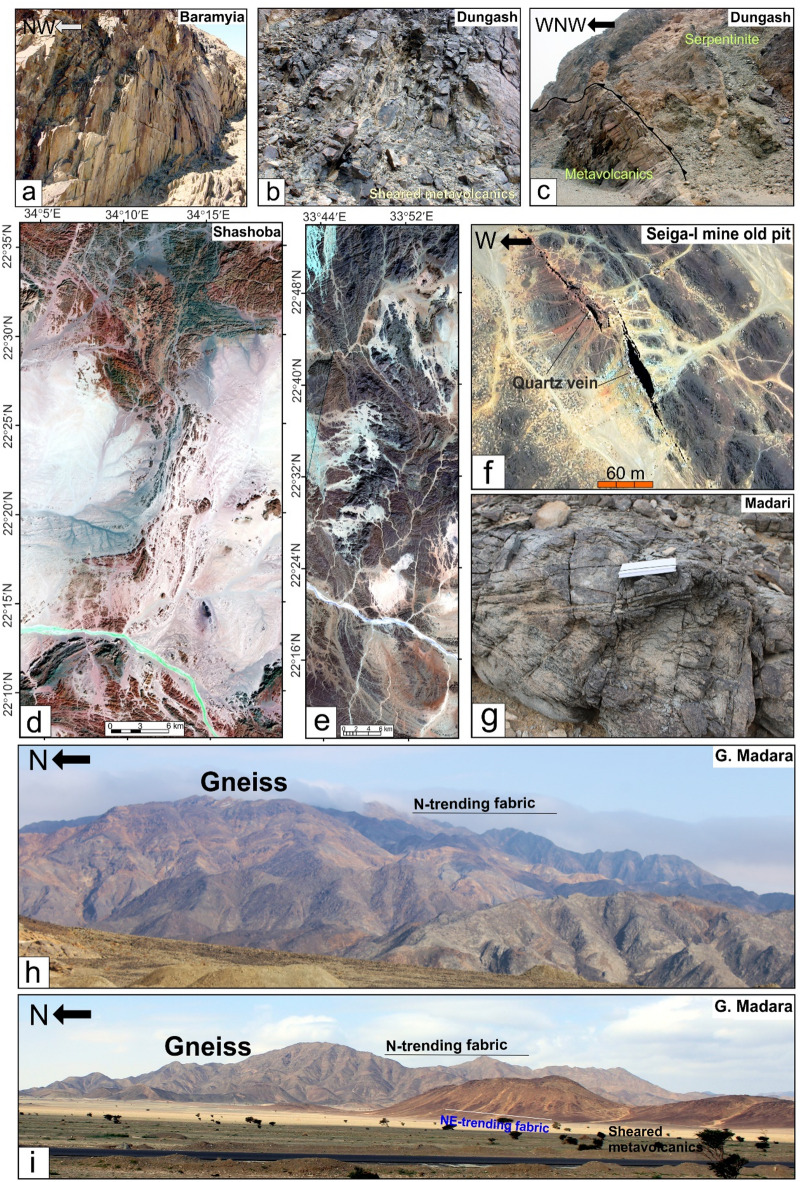
(**a**) N-S-striking subvertical foliation in volcanoclastic metasediments of Baramyia, (**b**) N-S shear fabric in metavolcanics of Wadi Dungash, (c) ENE-Dipping thrust between metavolcanics and talc-carbonate serpentinite along Wadi Dungash, (**d**) and (**e**) Landsat 8 true color composite images for the core of the Abu Swayel- Muqsim shear zone. The figures were created by ENVI v. 5.6.2. software; (https://www.l3harrisgeospatial.com/Software-Technology/ENVI), (**f**) NW-trending quartz veins in the Siga-1 mine old pit, (**g**) N-trending F3 fold in gneisses east of G. Madari (h) N-S-trending shear fabric in gneisses of G. Madari area, (**i**) N-S-trending shear fabric overprinted by NE-SW-trending foliation in G. Madari area.

The Barramiya-Mueilha region is characterized by increased S2 schistosity and mylonitic foliation that trends northwest. During the D3 deformation phase, the Barramiya-Mueilha shear zone emerged as a significant dextral shear zone oriented NNE (Fig. 14a **and b**), situated between metavolcanic and volcanoclastic metasediment in the area stretching from Wadi Dungash to Wadi Mueilha (Fig. 13a, b, **and c**). This shear zone exhibits a palm-tree-like configuration, with the central plane oriented NNE-SSW and steeply dipping towards the WNW^60^. The Wadi Beizah features imbricated thrust sheets composed of ophiolitic serpentinite (Fig. 14c) and metagabbro that have been subjected to open folding, resulting in a series of asymmetric, plunging synclinal and anticlinal folds trending NNW and measuring kilometers in scale (F3). The striking NE-SW to ENE-WSW (S4) cleavage has been superimposed on the earlier fabric and delineates the Um Saltite dextral shear zone. The most recent brittle deformation (D5) event includes WNW-ESE dextral and NNW-SSE sinistral strike-slip faults that have displaced all rock units (Fig. 13a, b, **and c**).

#### Abu Swayel- Muqsim shear zone (SMSZ)

The Abu Swayel-Muqsim region (Table 2) in Wadi Allaqi features three main litho-tectonic units: dismembered ophiolites, metavolcanic-metasedimentary sequences from an island arc, and granitoid intrusions linked to syn- and late-orogenic events^69,71–73,132,133^. Gneisses crop out in Wadis Abu Swayel, Haimur, and Um Ghalaga. In Abu Swayel, gneisses are interpreted as highly deformed metasedimentary rocks from a fore-arc basin^132,133^.

**Fig. 15:**
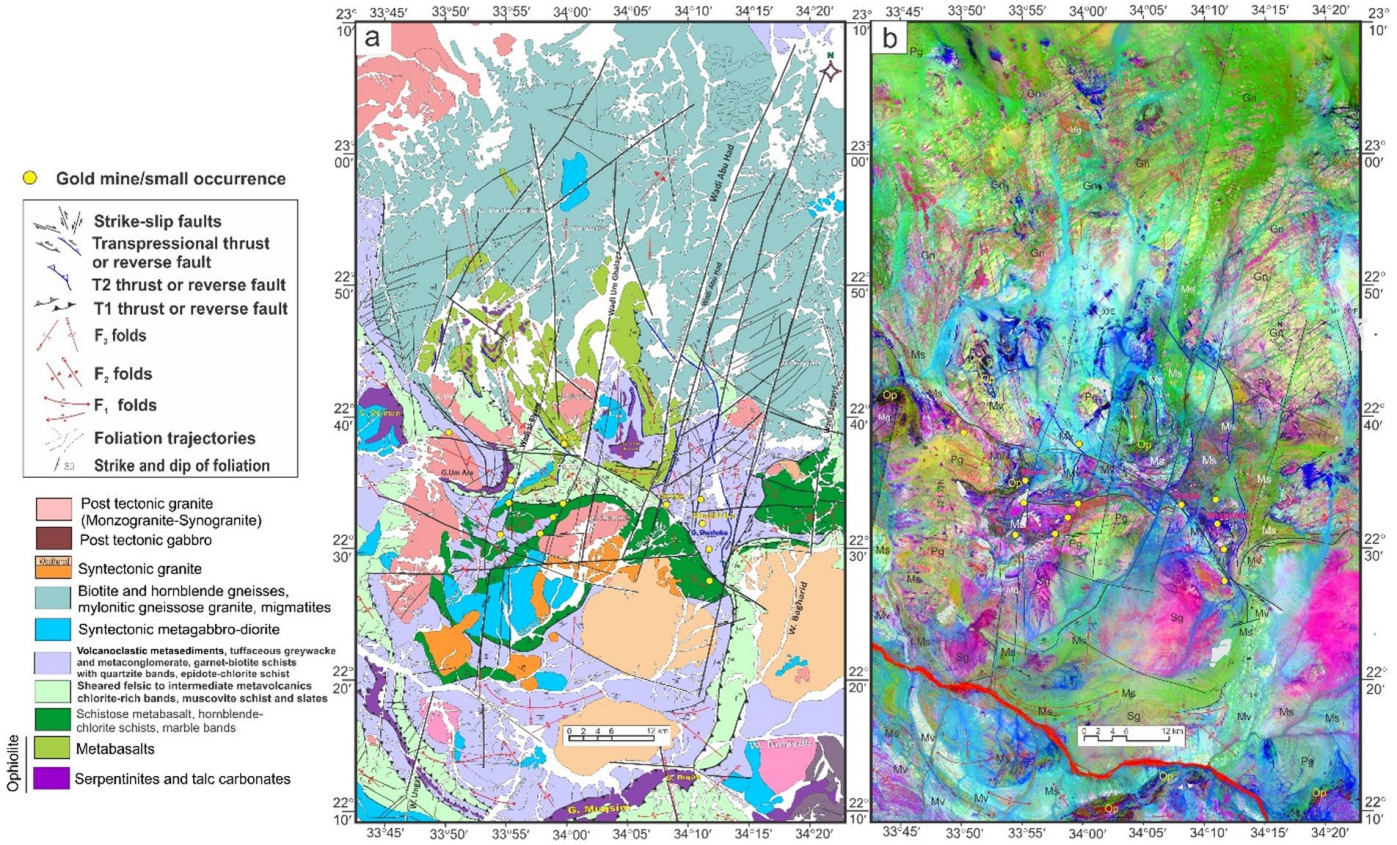
(**a**) Geological map of Abu Swayel- Muqsim shear zone (SMSZ) (compiled and modified after Conoco^129^; Zoheir et al.^131^; El Kazzaz^135^; Abd El-Naby and Frisch^132,133^; EGSMA^139^, (The figures were created by ArcGIS Desktop v 10.8. https://www.esri.com/en-us/arcgis/products/arcgis-desktop/overview) and SmartSketch v. 4.0 software; https://smartsketch.software.informer.com/4.0/). (**b**) PC653 in RGB of Landsat-8 showing the extension and major structures in Abu Swayel- Muqsim shear zone. (The figure was created by ENVI v. 5.6.2. software; (https://www.l3harrisgeospatial.com/Software-Technology/ENVI)

The chemical U-Th–Pb dating of monazites extracted from these gneisses indicates a peak metamorphic age of approximately 650–620 Ma^133^. The garnet-biotite and hornblende-biotite gneisses are located in the northern part of the Abu Swayel-Muqsim region, typically separated from the ophiolitic and island arc formations by strike-slip faults and thrusts. In the western and central areas of the Allaqi-Heiani suture, ophiolites are present as imbricate thrust-bounded sheets and slices of serpentinite, amphibolite, metagabbro, and metabasalt (Fig. 15a **and b**), all embedded within a highly tectonized talc carbonate matrix^71^. Within the study area, serpentinite appears as large mountainous blocks interspersed among tectonized ophiolites, metapelites, and marbles (e.g., G. Um Krush and G. Muqsum). The trace-element geochemistry of amphibolites from the Wadi Haimur–Abu Swayel region shows a transitional composition between mid-ocean ridge basalt (MORB) and island arc tholeiite (IAT), consistent with a back-arc tectonic setting^132^ .

Island arc metavolcanic formations from Gabal Shashoba to Wadi Um Ghalaga include basic to intermediate metavolcanics, intermediate to acidic metavolcaniclastics, and metatuffs (Figs. 14d and 15a **and b**). Basic metavolcanics are mixed with folded serpentinite and metasedimentary segments, with prominent N-trending folds^134^. West of Gabal Dheis and south of Gabal Um Krush, metavolcanics range from intermediate to acidic. Pelitic to psammopelitic metasediments, such as chlorite and garnet-chlorite schists, are intercalated with schistose metavolcanics and volcanoclastic metasediments (Fig. 14d**)**, often in fold closures and strain zones^69^. Marbles and quartzite bands are common in the western Allaqi-Heiani belt, near amphibolites along Wadi Haimur^135,136^.

The Gabbro-diorite complex is characterized by small outcrops (Fig. 15a **and b**). Syn-tectonic granitoids, predominantly quartz-diorite, tonalite, and granodiorite, are found in low-lying areas, where they intrude both older rock formations and younger granitoids. Additionally, late post-orogenic intrusions, primarily granodiorite and monzogranite, manifest as high- or moderately elevated stocks, appearing as sporadic, rounded or oval bodies (Fig. 14e) that penetrate most rock units.

In the region, quartz veins containing gold intersect altered metavolcanics and volcanoclastic metasediments, consistently influenced by shear zones trending NNE-SSW and NW-SE (Fig. 14f). Notable gold deposits in the area under investigation include the Shashoba, Seiga I (Fig. 14f), Seiga II, and Wadi Murra mines, as well as additional mining activities located north of Gabal Dheis and southwest of Gabal Um Krush^69^.

**Fig. 16:**
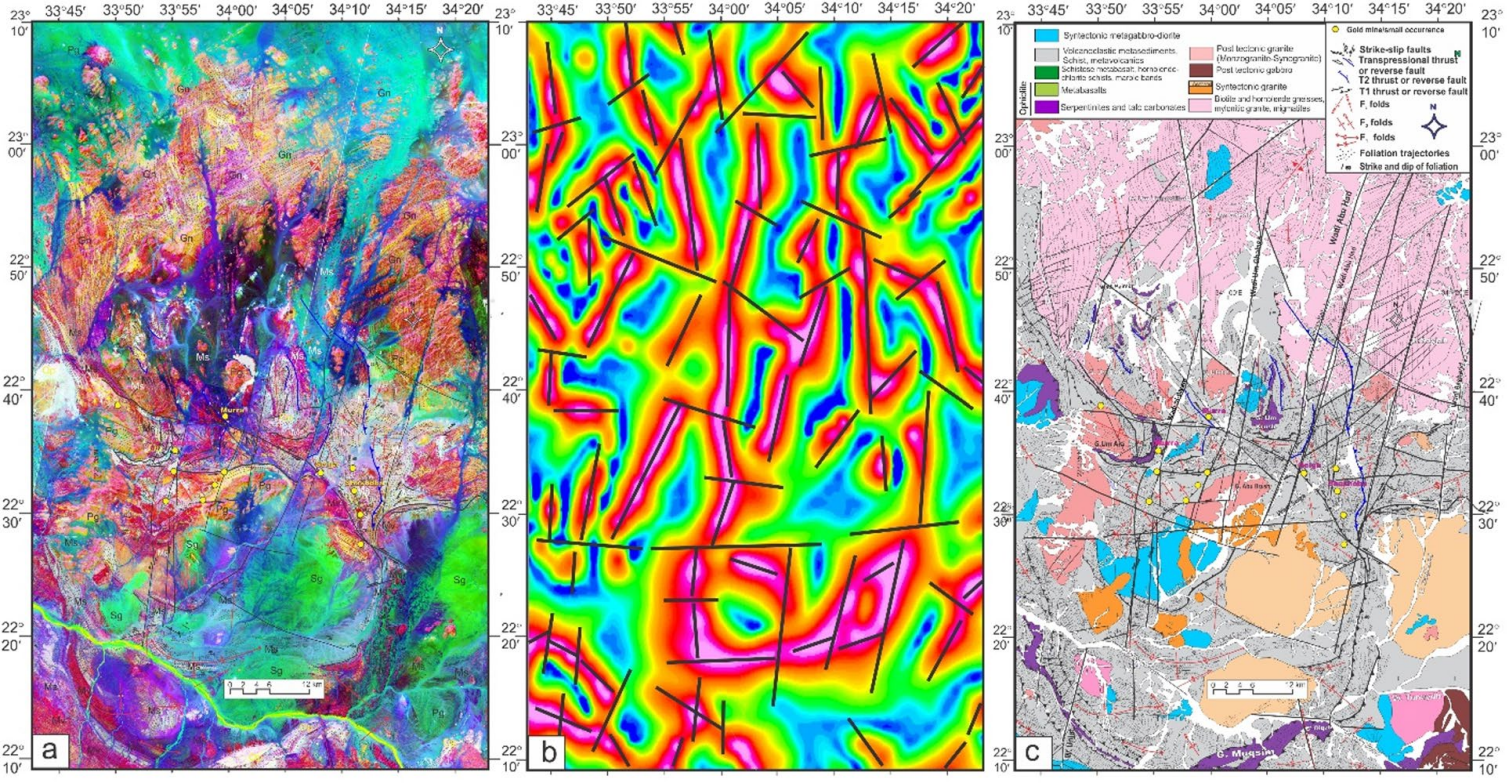
(**a**) MNF 2, MNF3, MNF4 in RGB showing the extent and major structures along the Abu Swayel- Muqsim shear zone. The figure was created by ENVI v. 5.6.2. software; (https://www.l3harrisgeospatial.com/Software-Technology/ENVI), (**b**) The LTHG map of the Abu Swayel- Muqsim shear zone, (**c**) Structural map of Abu Swayel- Muqsim shear zone (modified after Conoco, 1987). The figure was created using ArcGIS Desktop v10.8. https://www.esri.com/en-us/arcgis/products/arcgis-desktop/overview, and SmartSketch v. 4.0 software; https://smartsketch.software.informer.com/4.0/).

The Abu Swayel-Muqsim shear zone influences the central region of the Allaqi–Heiani suture, characterized by a series of N-S-oriented dextral ductile shear zones (Figs. 14d and e and 16a and b, **and c**). This area extends from Wadi Bagharid and Gabal Shashoba in the east to Wadi Ungat, Gabal Murra, and Gabal Shilman in the west. The Allaqi–Heiani suture exhibits a complex deformational history and metamorphic processes, culminating in localized metasomatism along steeply dipping, NNW-trending shear zones. The LTHG map (Fig. 16b) of the SMSZ reveals that the E-W, WNW-ESE, N-S, and NNE-SSW directions are the prevailing trends, with minor traces of NW-SE and NE-SW. The structural development of the suture is divided into four primary phases: D1, D2, D3, and D4. Phases D1 and D2 are associated with early accretion and northward subduction along the Allaqi-Heiani suture, occurring approximately between 750 and 720 Ma, while D3 and D4 correspond to late, post-accretionary east-west compression between the eastern and western segments of Gondwana^20,75,137^.

The initial phase of crustal N-S shortening (D1) was characterized by the movement and thrusting of ophiolitic blocks from the north onto sequences of metavolcanic and metasedimentary rocks associated with an island arc (Fig. 16a, b, **and c**). The western Allaqi-Heiani belt formed due to the collision of the Gabgaba and Gerf terranes along a subduction zone that dips approximately northward^71^. Structural features from D1 include pervasive cleavage foliation (S1), mineral stretching lineation (L1), folds oriented toward the east and west-northwest (F1), significant thrust faults trending E-W to WNW-ESE, and imbricate slices of ophiolite (Fig. 16a, b, **and c**).

In NE-SW transpression and oblique convergence (D2), NW-plunging folds and NW-SE foliation (S2) overlay thrust fabrics in schistose metavolcanic and ophiolitic rocks, indicating D2 deformation. The imbricate thrust system from D1 was later penetrated by deformed granodiorite bodies in the southern study area, shifting the shortening to NE-SW. Although S2 exists, it is significantly degraded in the eroded, lowland granodiorite intrusion^69^.

The S2 foliation, characterized mainly by muscovite, epidote, and chlorite with a steep dip, is accompanied by the L2 lineation, defined by quartz and hornblende, which increases westward. F2 folds, with wavelengths of 5–10 km and strike lengths up to 16 km (Fig. 16a, b, c), show shear sense indicators of overthrusts toward WNW and ESE^69^. The axes and axial surfaces of F2 folds vary around the regional transport direction, likely due to flow disturbances during movement along T2 thrusts and sinistral reverse shear zones^69^. The L2 lineation probably parallels the transport direction, indicating a regional swing and a gradual shift toward sinistrally oblique thrusting.

The sinistral transpression along pre-existing WNW-oriented thrusts and numerous NW-SE shear zones is thought to result from ongoing tectonic transport of ophiolitic nappes from the southeast to the northwest^69^. Significant structural developments during the D2 phase include transpression along the regional WNW-oriented thrusts and the emergence of oblique-slip reverse shear zones (Fig. 16a-c). The activity along these shear zones occurs between Gabal Um Krush and Gabal Abu Brush, extending up to 30 km in the central region of the study area.

The E-W compressional regime and northward tectonic escape (D3) have formed S3 fabric along NNW-SSE thrust-dominated shear zones and thrust segments (Fig. 14d **and e**). Additionally, D3 structures include tight to isoclinal folds trending NNW and N, and strike-slip faults that overlie older geological structures. The NNW-SSE thrust-dominated shear zones (Fig. 14d **and e**) locally host auriferous quartz veins (Fig. 14f), likely the final ductile deformation event in the Abu Swayel-Muqsim region. The S2 foliation is only mildly preserved in syn-tectonic granitoids and is subsequently overprinted by S3 (Fig. 16a, b, **and c**). The rigid characteristics of the granitoid bodies may have caused the D3-deformed schistose rocks to wrap around these extensive granitoid formations. Prominent NNE- and NNW-plunging F3 folds are observed in gneisses along Wadi Biyam, while smaller F3 anticlinoria and synclinoria, ranging from meter to kilometer scales, are noted in the central part of the map (Fig. 16a, b, **and c**).

The SMSZ is characterized by minor N-trending dextral zones that form a sigmoidal pattern with a mainly N-S orientation. Its central plane extends for hundreds of kilometers and can be up to 10 km wide, though its width varies along its length due to multiple branching secondary shear zones. These smaller shear zones are typically 1–2 km wide. Evidence of dextral shear is also visible in entrainment features at map scale. The SMSZ follows a curved path, shifting from north-northeast to north-northwest, which aligns with the local thrust orientation. This shear zone is believed to result from reactivation of a previous thrust zone through dextral strike-slip shearing.

The study area has gold-bearing quartz veins and alteration zones hosted by D3 shear zones. The latest deformation, D4, was predominantly brittle and linked to exhumation and cooling following late-orogenic intrusions. A network of NNE-SSW left-lateral strike-slip faults displaces earlier structures, with less extensive WNW-ESE and ESE-WSW right-lateral faults also creating displacements (Fig. 16a, b, c).

#### Himitrah-Madari shear zone (HMSZ)

**Fig. 17:**
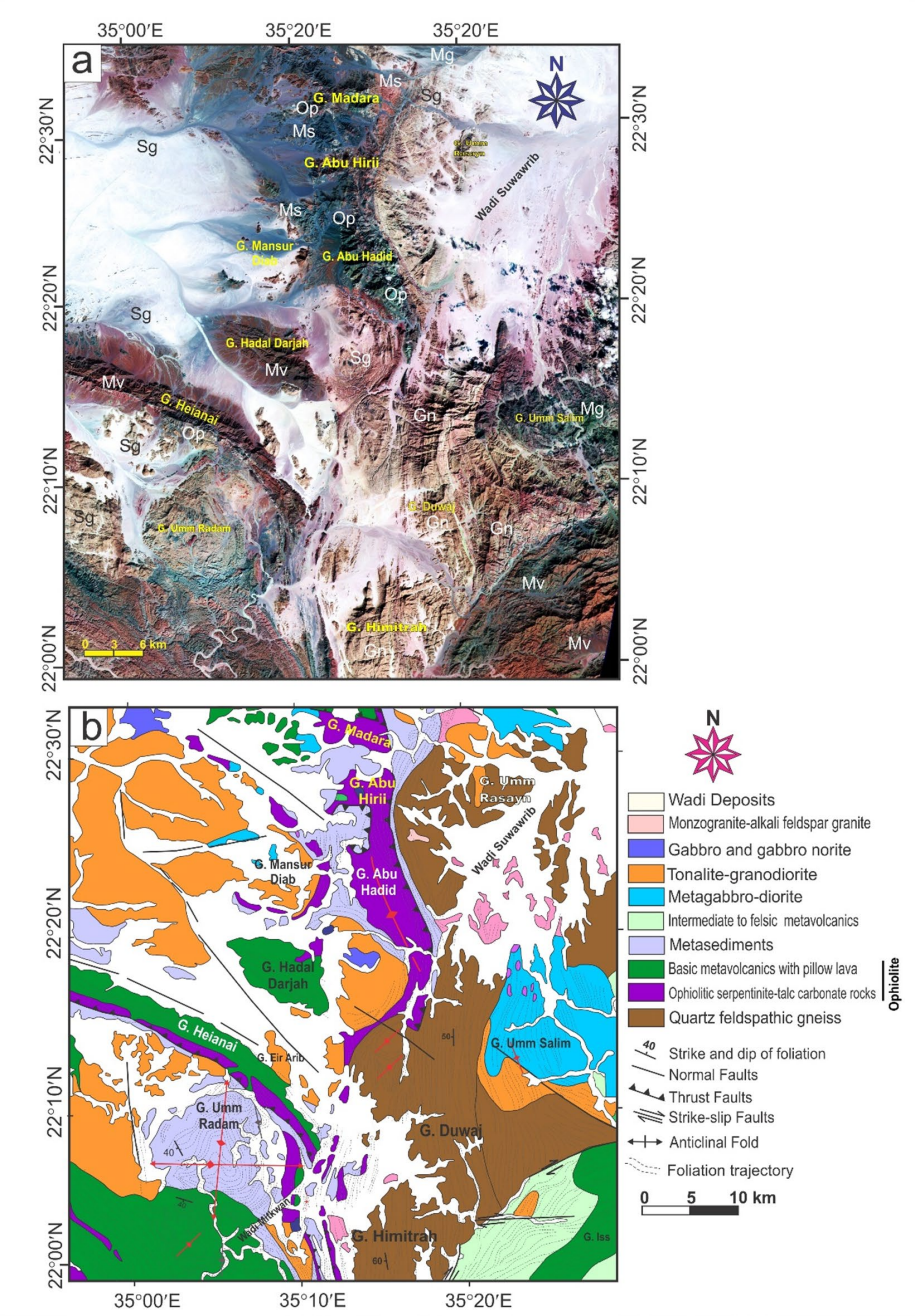
(**a**) True color Landsat 8 image (RGB 754) showing the Himitrah-Madari shear zone (HMSZ). The figure was created by ENVI v. 5.6.2. software; (https://www.l3harrisgeospatial.com/Software-Technology/ENVI), (**b**) Geological and structural map of Himitrah-Madari shear zone (Conoco^129^; Ali-Bik et al.^140^ EGSMA^141^. (The figure was created by ArcGIS Desktop v 10.8. https://www.esri.com/en-us/arcgis/products/arcgis-desktop/overview, and SmartSketch v. 4.0 software; https://smartsketch.software.informer.com/4.0/).

The NHSZ area (Table 2) comprises ophiolitic nappes, island arc-related metasediment-metavolcanic, and syn- and post-orogenic intrusions (Fig. 17a **and b**). Ophiolitic rocks occur as a structurally complex, elongate N–S trending belt in the western and southern part of the HMSZ area (Fig. 14g, h, **and i**), represented by gneisses, amphibolite, serpentinite, metagabbros, and highly tectonized ophiolites^140^. The gneisses exhibit a range of colors and well-developed foliation, with sharp contacts and alternating layers of leuco- and melanocratic rocks (Fig. 14h **and i)**. Serpentinites are the dominant rocks, forming high-relief, mappable lenses, ridges, and sheet-like bodies with steep slopes. Metagabbros, composed of actinolite, plagioclase, subordinate clinopyroxene, ilmenite, apatite, and epidote, are observed in the southern part of the NHSZ area and are thrust over both amphibolites and metasediments^79,140^. Amphibolites are found in limited locations in the western part of the area, near Bir Shani, and are formed thrust-bounded sheets intimately associated with serpentinites.

The island arc assemblage in the NHSZ region consists of layered metasedimentary and metavolcanic rocks. The metasediments create a low- to moderate-elevation landscape and are unconformably covered by ophiolitic rocks, which are thrust over metavolcanics^80,140^. These rocks exhibit high foliation, including biotite schists and mineralized quartzite bands (Fig. 17a **and b**). The mineralized quartzites are dense, rugged, and uniform in texture, composed of fused quartz sand grains. Metabasalts, displaying varying degrees of schistosity and forming an N–S trending belt at the core of the HSZ, are tectonically overthrust by the metasediments.

The intrusive rock assemblage includes syn- and post-tectonic intrusions, mainly granodiorites (Fig. 17a **and b**). Syn-tectonic intrusions are coarse-grained, enriched in mafic minerals, and mainly granodioritic. They cover extensive areas in the southern part of the study area and occur as sharp N-S-oriented ridges, particularly along the HSZ trace.

Post-tectonic intrusions display no foliation and intrude the island arc and ophiolitic rocks. They are distributed throughout the study area, occurring commonly in small exposures, particularly in gabbros and alkali-feldspar granites^80,140^. Post-tectonic granites’ intrusive phases are characterized by muscovite granites and alkali feldspar granites (Fig. 17a **and b**). Muscovite granites are fine-grained to coarse-grained and occasionally pegmatitic. The alkali feldspar granites intruded the ophiolitic and syn-tectonic granite rocks.

**Fig. 18:**
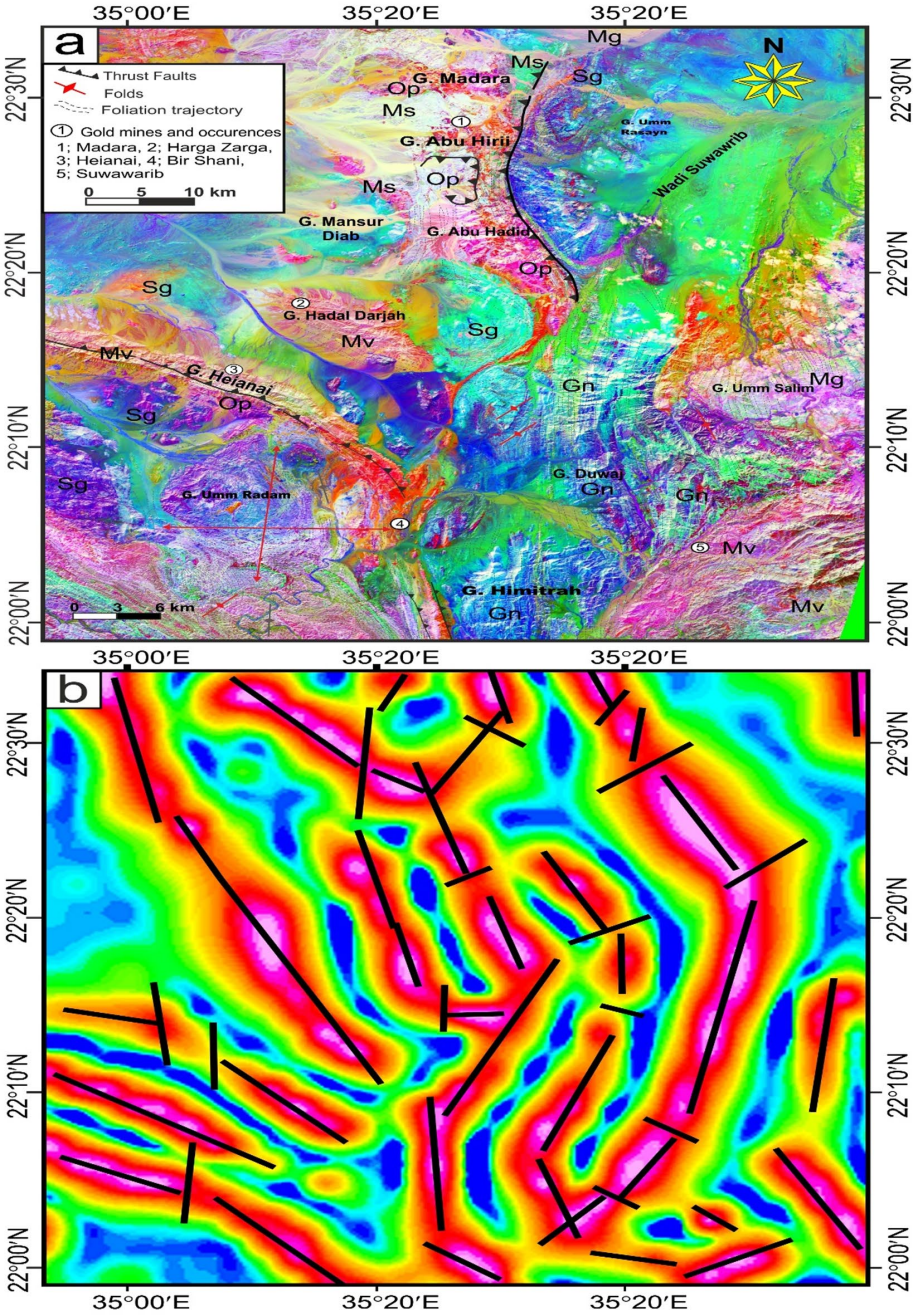
(**a**) MNF 2, MNF4, MNF5 in RGB showing the extend and major structures along the Himitrah-Madari shear zone, (The figure was created by ENVI v. 5.6.2. software; (https://www.l3harrisgeospatial.com/Software-Technology/ENVI), (**b**) The LTHG map of the Himitrah-Madari shear zone. The figure was created by Geosoft Oasis montaj v. 8.3.3. (https://www.seequent.com/products-solutions/oasis-montaj/).

Field observations and satellite images have identified four deformational events in the Neoproterozoic rocks of the Himitrah-Madari area (Figs. 17b and 18a). The LTHG of GBG data of the HMSZ (Fig. 18b) indicates that the area is affected by WNW-ESE, NNE-SSW, NW-SE, and NNW-SSE orientations. D1 is an early N-S shortening, caused by early E-W thrusting and folding (F1) that impacted ophiolitic and island-arc-related metasediments. D2 features NW-trending structures formed under the ENE-WSW compressional regime, recorded in ophiolitic and island-arc rock assemblages. The third event, D3, involves E-W compression and the development of N-S-striking mylonitic foliation and N-trending folds, creating the Himitrah-Madari shear zone (HMSZ) (Fig. 14g, h, **and i**). The shear zone is the northern extension of the Hamazana shear zone, which extends about 300 km in Sudan. D4, an extensional event, is seen as a post-orogenic extensional phase characterized by E-W dextral strike-slip and dip-slip normal faults striking NNW-SSE to N-S and E-W, occurring after the emplacement of post-tectonic granites. This event is linked to post-orogenic processes related to Red Sea rifting. Folding is mainly localized around and/or associated with the thrust belt sheet (Figs. 17b and 18a), especially within the island-arc assemblages. Several upright, overturned, and recumbent folds at various scales are documented in the study area.

Three distinct folding events were identified: F1, F2, and F3. F1 (E–W) folds are mainly limited to ophiolitic metagabbro and island-arc metasedimentary rocks near and associated with the E–W thrust faults. F1-folding events exhibit steeply dipping to vertical north-dipping and, less frequently, south-dipping planes. F2 (NW–SE) folds are linked with NW-thrust faults in ophiolitic rocks and gneiss (Figs. 14g and 17b, and 18a), as well as in island-arc metasediments and metavolcanics. These folds trend NW, are asymmetric, recumbent, and tight, with kinked geometries, and feature subhorizontal axes that gently plunge toward the NW or SE. Mineral lineations (L2) are aligned parallel to the fold axes and gently plunge toward the NW or SE. F3 (N–S) folds are widely observed in the HMSZ area, ranging from open at the kilometer scale to tight, upright, and slightly asymmetric (Figs. 17b and 18a). The wavelength of these folds decreases toward the high-strain zone of the HSZ, indicating a progressive increase in strain. This increased strain coincides with the steepening of the S3 foliation toward nearly vertical and the formation of mylonite schist. The F3-folding event predominantly involves steeply to vertically dipping E-W-oriented planes that gently plunge toward the north and south. Generally, sub-horizontal to moderately plunging N–S lineations (L3) are well developed within the metasediments and metavolcanics.

## Discussion

### Kinematics of the N-S shear zones

**Fig. 19:**
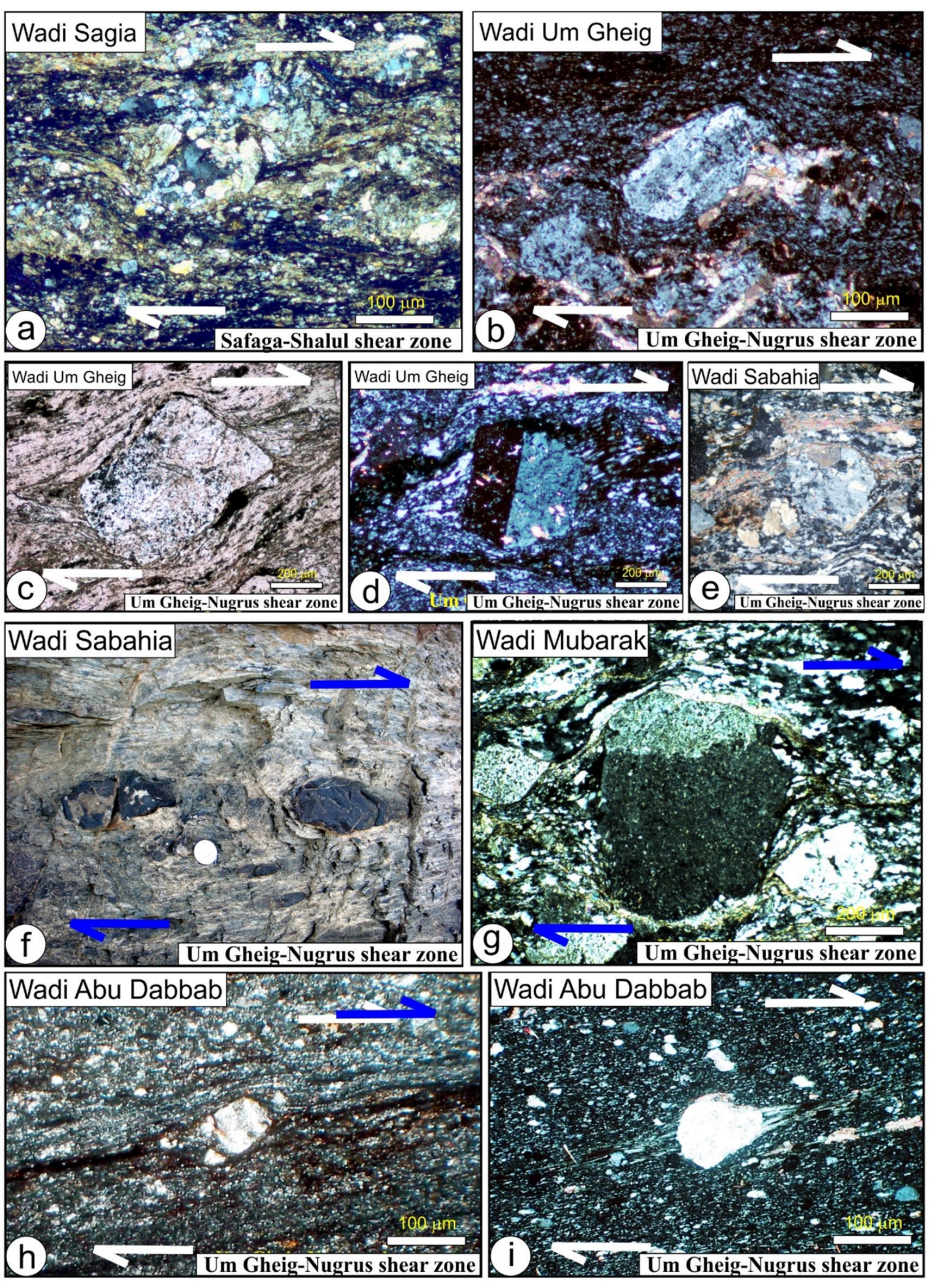
Shear sense indicators with dextral sense of shear along the N-S-striking ductile shear zones. (**a**) Augen-shaped quartz porphyroclasts in porphyroclastic mylonite, Wadi Sagia, Safaga-Shalul shear zone, (**b**–**d**) Augen-shaped plagioclase porphyroclasts in mylonite schist, Wadi Um Gheig, Um Gheig-Nugrus shear zone, (**e**) Augen-shaped Quartz porphyroclasts mylonitic gneiss of Wadi Sabahia, Um Gheig-Nugrus shear zone, (**f**) Ultramafic clasts in mylonitic schist, Wadi Sabahia, Um Gheig-Nugrus shear zone, (**f**) Augen-shaped plagioclase porphyroclasts in prophyroclastic mylonite, Wadi Mubrark, Um Gheig-Nugrus shear zone, (**g**–**i**) Augen-shaped quartz porphyroclasts in prophyroclastic mylonite, Wadi Abu Dabbab, Um Gheig-Nugrus shear zone.

**Fig. 20:**
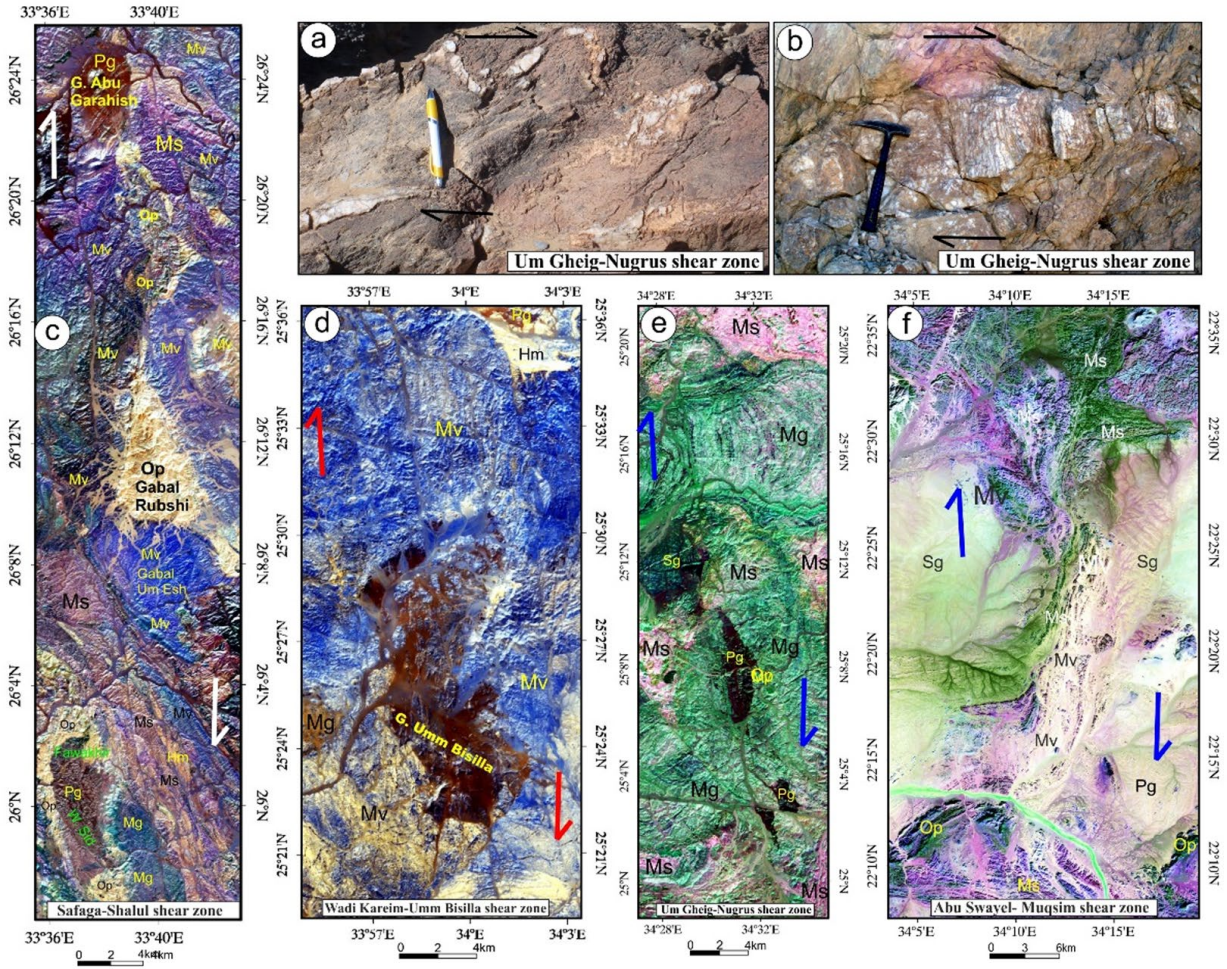
Folded quartz veins, (**a**) and augen shaped quartz (**b**) in Baramiya-Dungash shear zone, indicating dextral sense of shear. Oblique foliation and deflection and warping of foliation around serpentinite and granite masses indicating dextral sense of shear in (**c**) Band ratios of 4/6, 1/7, 2/5 for the core of Safaga-Shalul shear zone, (**d**) Band ratios of 4/7, 3/7, 3/5 for the central part of Wadi Kareim-Umm Bisilla shear zone, (**e**) Band ratios of 5/2, 6/4, 1/3 for the central region of Um Gheig-Nugrus shear zone and (**f**) Band ratios of 7/1, 5/3, 6/3 the southern part Abu Swayel- Muqsim shear zone. The figures were created by ENVI v. 5.6.2. software; (https://www.l3harrisgeospatial.com/Software-Technology/ENVI)

Shear-sense indicators and stretching lineations play a crucial role in the kinematic analysis of shear zones, providing essential insights into the dynamics of movement within these geological structures. Researchers typically analyze the orientation of these lineations to determine the shear direction. A horizontal lineation usually indicates strike-slip movement, whereas an oblique lineation points to oblique-slip movement. In ductile shear zones aligned in an N-S orientation, the asymmetrical features observed in mylonitic rocks indicate non-coaxial strain associated with the shear overprint from the N-S shear zones. A diverse array of kinematic signatures includes both macro- and microscopic S/C fabrics, shear bands, and mantled porphyroclasts that display σ- and occasionally δ-shaped stair-stepping tails (Fig. 19a, b, c, d, f, g, h, **and i**). Other diagnostic features include volcanically folded quartz veins (Fig. 20a) and augen-shaped quartz veins (Fig. 20b), inclined shear-related folds, and deformational disruption to orientations of foliation around locations of serpentinite and granite (Fig. 20c, d, e, f). The analysis indicates that, taken together, these data suggest a dextral shear sense (Fig. 19) within the N-S-oriented shear zones of the ENS.

Additionally, the brittle N-S strike-slip faults reflect sinistral displacement. The horizontal displacement of rock layers, such as molasse sediments, schistose metavolcanics, metagabbro, and post-tectonic granites, represents a significant structural feature that corroborates sinistral movement. The kinematic history of the N-S shear zones indicates that they originated as large-scale dextral ductile shear zones overprinted by brittle sinistral strike-slip faults.

### Deformation history of the N-S shear zones

**Fig. 21:**
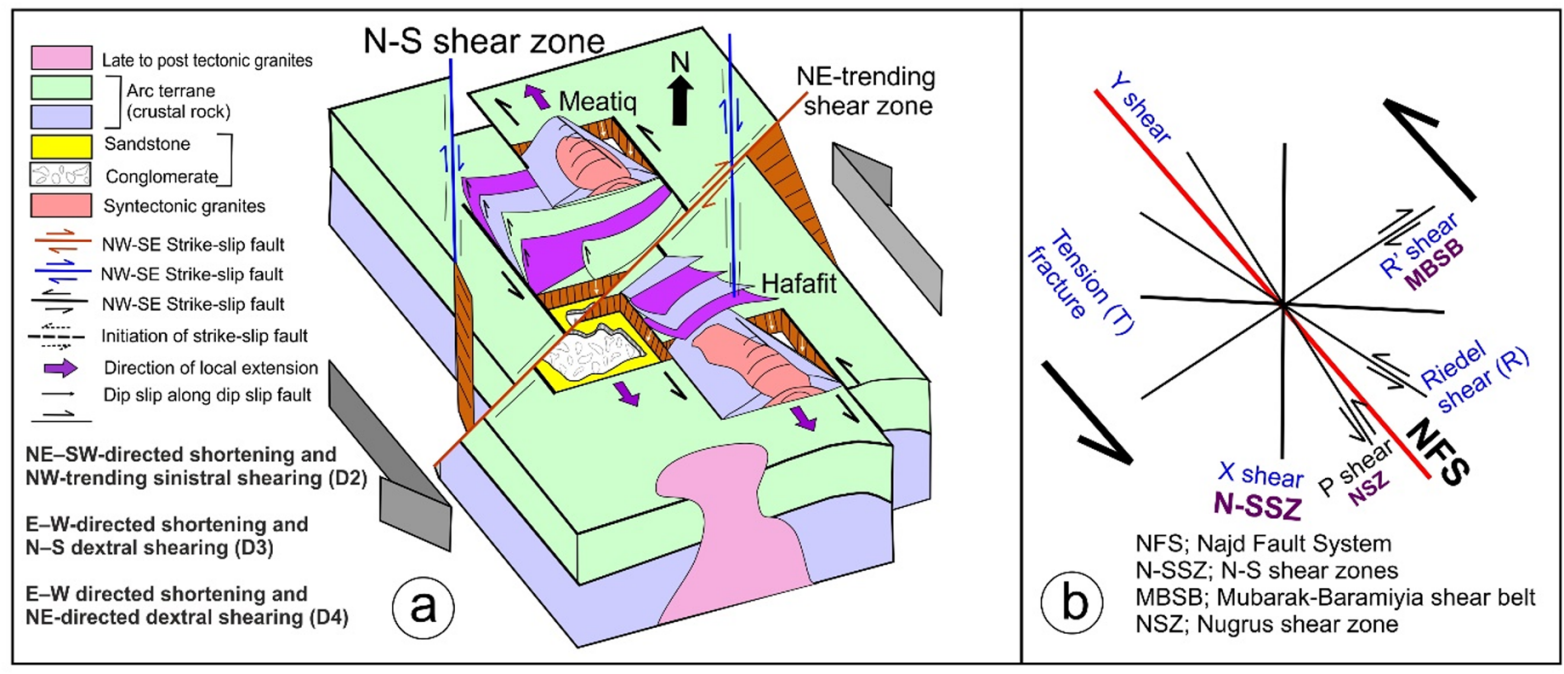
Schematic diagrams (modified after Abu Sharib^151^) illustrate how alternating zones of compression and extension can form between convergent and divergent strike-slip shear zones during shearing along the Najd Fault system and development of NW-trending sinistral shear zones (D2), N–S dextral shear zones (D3), and shear zones with NE-directed dextral shear zones (D4). These zones of compression and extension develop into thrusts and pull-apart structures, respectively. Continued wrenching may result in the creation of pull-apart basins and the exhumation of structurally lower metamorphic core complexes. The figures were created by ArcGIS Desktop v 10.8. https://www.esri.com/en-us/arcgis/products/arcgis-desktop/overview, and SmartSketch v. 4.0 software; https://smartsketch.software.informer.com/4.0/).

Integrating field observations with magnetic and remote-sensing analyses enables us to categorize the structural development of the N-S shear zones into five primary deformational stages. These stages include: (1) the N-S shortening phase characterized by southward thrust imbrications (D1), which is marked by a nearly N-S compression evident in south-verging thrust segments and the formation of E-W mineral foliations and folds within ophiolitic and volcano-sedimentary rock formations; (2) a phase of NE–SW-directed shortening (Fig. 21) and NW-trending sinistral shearing (D2), representing a simple shear phase that resulted in the formation of kilometer-scale northwest and north-northwest sinistral faults; (3) E–W-directed shortening accompanied by N–S dextral shearing (Fig. 21), which led to the development of shear zones striking N-S, NNW-SSE, and NNE-SSW; (4) E–W-directed shortening and NE-directed dextral shearing (D4), which produced ENE-WSW and NE-SW dextral shear zones (Fig. 21), contributing to a three-component system within a significant NW-strike-slip sinistral shear system, likely associated with the Najd shear system; and (5) the brittle strike-slip faults stage (D5), characterized by terrain uplift, extension, Red Sea rifting, and the formation of various dykes and barren quartz veins, alongside the establishment of a transcurrent shear system that overlapped existing discontinuities, leading to the reactivation of earlier shear zones. Table 2 outlines the orientations, ages, and structural styles of significant sutures, syn- and post-accretionary structures within the Egyptian Nubian Shield.

#### The N-S shortening phase and southward thrust imbrications (D1)

This event marks an initial collision phase between the South Eastern Desert terrane to the north and the Haya and Gabgaba terranes to the south, approximately between 830 and 720 Ma. This period is also associated with the development of the Yanbu–Sol Hamed–Gerf–Allaqi–Heiani arc–arc suture^6–8,15,20,26,67,71–73^. Deformation during this phase is primarily localized in the Compressional Domain, characterized by suture development from terrane accretion around 750–720 Ma. The Yanbu–Sol Hamed–Gerf–Allaqi–Heiani arc–arc suture extends eastward from the entrance of Wadi Allaqi to Lake Nasser, following the boundary between Egypt and Sudan, and concludes at Gabal Gerf near the Red Sea^14–16,22–25,80^.

**Fig. 22:**
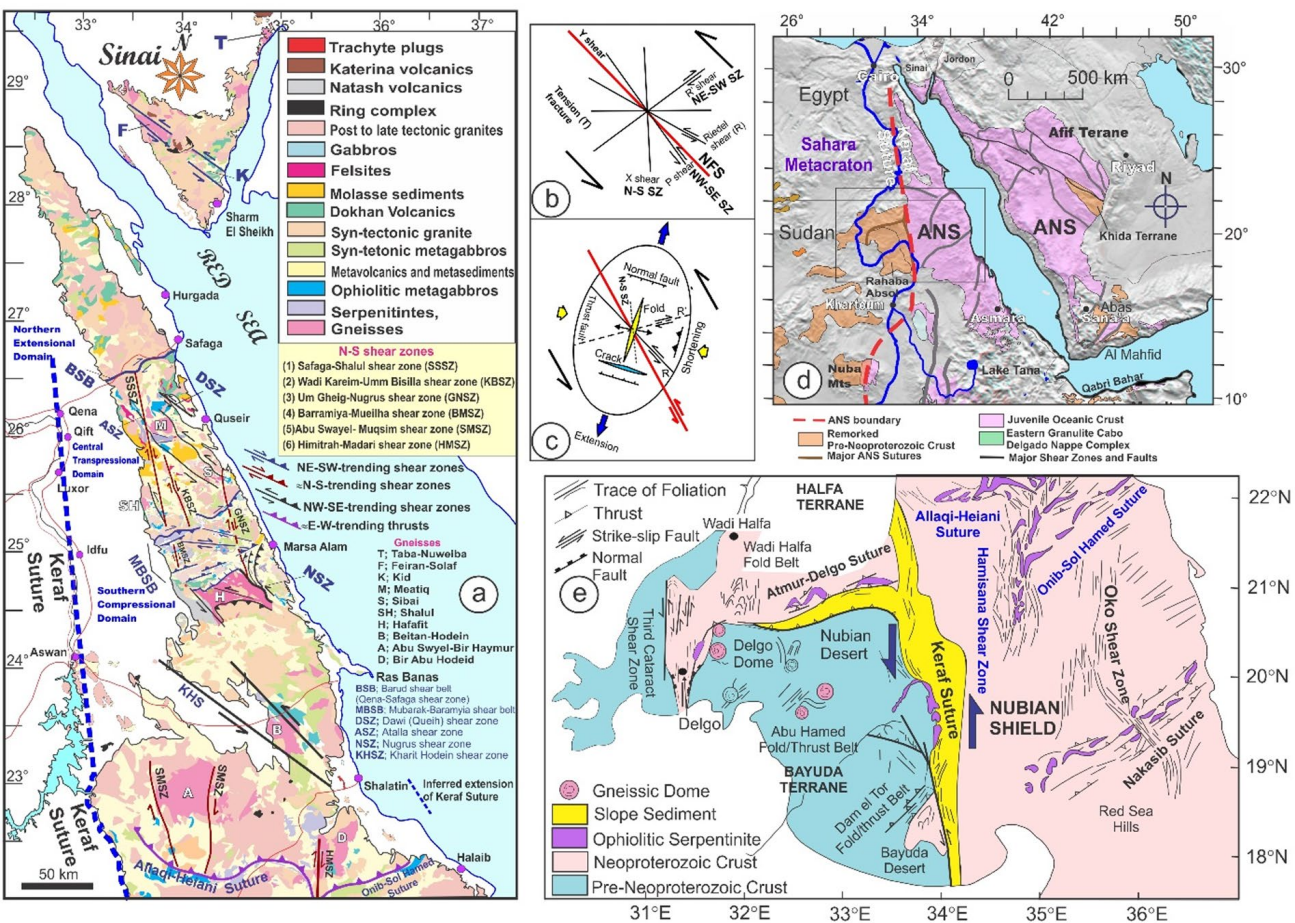
(**a**) Geological map of the Egyptian Nubian Shield showing the major structures in relation to the N-S-trending shear zones, (**b**) and (**c**) Riedel structures and strain ellipsoid showing relation between NW-SE, N-S and NE-SW-striking shear zones in the ENS, (**d**) Distribution of crustal domains in the northern part of the East African Orogen (EAO) showing the relation between the Arabian-Nubian Shield (ANS) and the Keraf suture (adapted from Abd El Wahed and Attia^15^, (**e**) The southern segment of the Nubian Shield showing the attitude of the Keraf suture zone in relation the Hamisana shear zone, Onib-Sol Hamed suture and Allaqi-Heiani suture (adapted from Abdelsalam et al.^71^). (The figures were created by ArcGIS Desktop v 10.8. https://www.esri.com/en-us/arcgis/products/arcgis-desktop/overview, and SmartSketch v. 4.0 software; https://smartsketch.software.informer.com/4.0/).

The Allaqi shear belt region underwent considerable N-S shortening, leading to the development of east-west foliation (Fig. 22a) in ophiolitic rocks and volcanoclastic metasediments. The initial S1 structure in the Abu Swayel-Muqsim shear zone is distinguished by banding and schistosity in volcanoclastic metasediments, sheared metavolcanics, mafic schists, and metagabbros. The D1 structural characteristics are defined by thrust imbrications that initially dip to the north but later shift to NW-SE and NE-SW orientations during the D2 phase (Fig. 22a). These thrusts delineate the boundaries between the ophiolite slices and the adjacent volcanoclastic metasediments and metavolcanics. Furthermore, very tight to nearly isoclinal folds, oriented west-northwest to east-southeast, in the volcanoclastic metasediments and schistose metavolcanics indicate earlier D1-linked folding events. The orientation of these thrusts varies from west-northwest to east-southeast and from northwest to southeast, effectively establishing the interface between the ultramafic rocks in the hanging wall and their significantly sheared derivatives found in the footwall, which consist of metasediments, metavolcanics, and metatuffs. These thrusts are aligned with the primary foliations and axial traces of the F1 and F2 folds. They are characterized by cataclastic features and pronounced shearing, as indicated by the narrow mylonite zones.

#### NE–SW-directed shortening and NW-trending sinistral shearing (D2)

The second event (D2) is characterized by the formation of structures that emerged under an NE-SW compressional regime. This phase is the initial stage of the second collision episode within the Pan-African orogeny. East and West Gondwana converged, compressing the Arabian Nubian Shield. This NE-SW compressional event significantly affects the Central Eastern Desert and the northern section of the Southern Eastern Desert, particularly through the development of NW-trending sinistral shear zones associated with the Najd Fault System (NFS). The Transpressional Domain in the ENS is characterized by the Najd Fault System (NFS), a complex array of NW-SE orientated sinistral strike-slip ductile shear zones established between 540 and 620 Ma^34,35^.

The continuous NE–SW-directed compression has resulted in the refolding of nappes, which are distinguished by steeply inclined, NW-oriented folds (Fig. 21). The D2 phase is chiefly characterized by folded thrusts and mylonite zones delineating the boundaries between ophiolitic slices, metavolcanics, and volcanoclastic metasediments (Figs. 21 and 22a). The regional S2 planar fabric shows considerable folding around gently plunging NW-SE axes. Numerous NW-trending mesoscopic folds occur within the volcanoclastic metasediments, as well as in both felsic and mafic schists, and in sheared metavolcanics. The mesoscopic and macroscopic folds identified in the central area of the ENS, from Gabal Gerf in the south to the Queih shear zone in the north, range from gentle to tight and include asymmetrical, recumbent, chevron, and overturned styles. The dominant axial planar structure is characterized by crenulation cleavage (S2), which trends northwest and dips moderately to steeply toward the NE or SW. The axial traces of these geological formations exhibit considerable variation, ranging from 1 km to millimeters. In the northwest-trending shear zones, the rocks have experienced deformation, developing mylonites and ultramylonites characterized by a prominent mylonitic foliation and sigmoidal porphyroclasts indicative of sinistral shearing. The abundance of mylonites in regions of intense strain is apparent, frequently manifesting as banded schists.

The Transpressional Domain and the northern section of the Compressional Domain are predominantly defined by an NW-trending tectonic structure (Fig. 21a), which delineates the NW-SE sinistral shear zone of the Neoproterozoic Fold and Shear^30,31,60,61^. The D2 phase is characterized by folding, thrusting, and the subsequent development of the NFS^32,66,85,142–145^. Thrust-related formations are linked to the oblique convergence of the arc and back-arc assemblage onto the Saharan Metacraton, occurring approximately between 620 and 640 Ma^145^. Following this, the northwest-trending sinistral shear zones of the NFS emerged^30,32,37,38,58,59^. This deformation phase was marked by transpression and lateral extrusion, leading to the exhumation of core complexes in an orogen-parallel extension between 620 and 580 Ma^2,15,30,65–67,116^. The sinistral strike-slip shearing along the NFS occurred alongside transpressional and transtensional tectonic environments^30^. According to Abu-Alam et al.^142^, all structural events in the northern Arabian–Nubian Shield over the last 90 million years of the Pan-African orogeny were classified as part of the NFS. This classification was initiated in a compressional tectonic context characterized by strike-slip activity, eventually leading to escape tectonics. The most significant NW-trending sinistral shear zones that formed in the ENS during the D2 stage include the Kharit-Hodein shear zone, Nugrus shear zone, Atalla shear zone, and the gneiss domes associated with these shear zones.

#### E–W-directed shortening and N–S dextral shearing

In the D3 phase, the initial E-W and NW-oriented folds, NE-dipping thrusts, and NW-trending shear zones were deformed into N-trending folds due to dextral shearing along N-S shear zones (Fig. 21). This period saw the formation of several N-S-trending shear zones (Fig. 22a), which have an estimated age of 600-590 Ma^6^, affecting all rock units except for the post-tectonic granite. The early structures in the ENS evolved into macroscopic N-trending folds and a network of mylonitic shear zones striking N-S (Fig. 21). The strain appears to have shifted from a dominant N-S shortening, linked to the emplacement of nappes, to a more E-W-directed shortening, resulting in the creation of the initial N-trending folds, steeply dipping axial planar foliation (S3), and N-S-trending dextral strike-slip shear zones. The overlap of N-trending folds on earlier SW-verging folds has produced a crescent-shaped dome interference pattern characterized by distinct rock ridges.

Analysis of the N-S shear zones shows that the N-SSZ underwent simple shear deformation as it shifted from ductile to brittle-ductile conditions (D3). The structural setup includes linear shear zones 50–150 km long and 3–10 km wide, with steeply N-S-dipping fabrics (S3) and both subhorizontal and moderately plunging stretching lineations. At the outcrop level, structures are mainly identified by mylonitic and shear foliation and subhorizontal lineations. These zones contain rock units with varying degrees of deformation, ranging from highly deformed volcaniclastic metasediments and Hammamat sediments to minimally deformed metavolcanic, metagabbro-diorite, and synorogenic granites. The cores of the shear zones show intense deformation, with prominent N-S stretching and folding, and shearing increases significantly near ductile shear zones.

#### E–W directed shortening and NE-directed dextral shearing (D4)

A prominent NE-SW to ENE-WSW trending shear fabric (S4) characterizes the ENS, characterizing two main shear belts (Figs. 21 and 22a): the Barud shear belt (part of the Qena-Safaga shear zone) and the Baramiya-Mubraka shear belt (part of the Idfu-Marsa Alam shear zone). The early-developed structural fabrics exhibit refolding around NE-to-ENE-trending axes, with a notable absence of structures trending to the north and northwest. The Barud shear belt has a succession of discontinuous, curved splays extending several kilometers in width and trending northeastward. The splay intersects the synorogenic granites in Wadi El Bula and Wadi Safaga. The eastward movement of the Barud shear zone induces deformation in the metagabbro, amphibolite, and hornblende schist. The northern segment of the Safaga-Shalul shear zone is overprinted by the NE-SW shear fabric linked to the Barud shear zone.

In contrast, the southern segment of Safaga-Shalul and Wadi Kareim-Umm Bisilla shear zones is overprinted by the NE-SW shear fabric of the Baramiya-Mubraka shear belt. The um Gheig-Nugrus shear zone traverses the Baramiya-Mubraka shear belt, forming a conjugate shear fabric. The Mubarak-Barramiya Shear Belt (MBSB) is an enormous NE-trending high-strain belt that extends from Wadi Mubarak on the Red Sea Coast to the area west of Barramiya. MBSB is marked by sheared ophiolite slices scattered in schistose mélange, which separates low-grade volcanogenic metasediments and metavolcanic in the north from the medium-grade gneissic migmatites of Hafafit dome.

#### Brittle strike-slip faults (D5)

This event pertains to the uplift of the terrain and the spreading of the Red Sea. The ductile N-S dextral shear zones have been reactivated as brittle sinistral strike-slip faults. A key example is the Um Gheig-Nugrus shear zone, which sinistrally displaces the Kadabbora granite pluton along the NE-SW trending Baramiya-Mubraka dextral shear belt. Additionally, the Safaga-Shalul shear zone has been rejuvenated into sinistral strike-slip faults, affecting all rock units, including G. Abu Garahish, G. Kab Amiri, and the Atalla shear zone. The brittle strike-slip faults found in the ENS are classified into four key orientations: N-S (N–S), NW-SE (NW–SE), NE-SW (NE–SW), and east-west (E-W). The NW–SE and N–S faults are characterized by sinistral displacement, whereas the NE–SW and E-W faults show right-lateral displacement. The map distinctly highlights the NE–SW and E-W fault systems, intersecting with the dextral N–S faults that offset the left-lateral NW–SE strike-slip faults.

### Structural Characteristics of the N-S shear zones in the Egyptian Nubian Shield

The N-S-oriented dextral shear zones within the ENS are characterized by several key structural features: (i) form complex system of ductile shear zones that demonstrate a dextral sense and are arranged in an en echelon structural style; (ii) these shear zones are enriched with ophiolite materials, consisting of highly deformed ophiolite nappes, metavolcanic rocks, volcaniclastic metasediments, and intrusive rocks; (iii) the occurrence of belts and antiforms of mylonitic schist exhibiting more significant deformation compared to the surrounding lithologies (iv) they extend for hundreds of kilometers and can reach widths of up to 10 km, making them distinctly visible in the Egyptian Nubian Shield, where they are associated with transpressional belts; (v) the identification of gently N- and S-plunging mineral and stretching lineations, alongside E-moderately plunging lineations; (vi) the deformation of various rock types, including syn- to late tectonic granites, Hammamat molasse sediments, and felsite, where syn-tectonic granitoids develop mylonitic granite along the fault zone. and (vii), they influence the E-W (D1) and NW-SW (D2) structural fabrics, which are further overprinted by a NE-SW (D4) shear fabric. The shear zones oriented NW-SW, N-S, and NE-SW in the ENS are conjugate shear zones formed during a unified progressive deformation event tied to the NFS’s activity. They have since been rejuvenated as brittle sinistral strike-slip faults.

The conjugate shear zones are located in the central transpressional domain and exhibit a combination of NW-trending sinistral and NE-trending dextral transpression, which originated during the Najd orogeny. The dextral shearing aligned in a N-S direction overlays the pre-existing NW-trending shear fabric and is further superimposed by NE-trending dextral shearing. The NW-SE shear zone represents the dominant shear plane, while the NE- and N-S-trending shears act as conjugate structures. Riedel structures, or R shears, appear as networks of shear bands that typically develop in simple shear zones during the early stages of faulting, as described by Katz et al.^146^. In this framework, the NW-trending sinistral shear is identified as the primary shear plane (Y) (Fig. 22b **and c**), the NE-trending dextral shear is associated with the R’ shear (Fig. 22b **and c**), and the N-S dextral shear is classified as the X-shear (Fig. 22b **and c**).

Geophysical investigations indicate that these primary shear zones extend beneath the surface rocks, implying a potential association with the extensive N-S striking shear zones in the Arabian Nubian Shield, including the Hamazana shear zone and the Keraf suture. The Hamazana shear zone and the Keraf suture represent a major north-trending megashear within the Nubian Shield. The Hamisana Shear Zone (HSZ) is a prominent high-strain area in Sudan, covering approximately 15,000 km² and constituting one of the most significant N-S-oriented basement structures in northeastern Africa. Situated between the Gabgaba Terrane and the Gebeit Terrane, it is identified as a suture zone marked by either strike-slip displacement or crustal shortening^15,74,75^. The HSZ is a post-accretion suture linked to the Najd fault system (NFS) that formed as the Arabian-Nubian Shield underwent shortening between 650 and 550 Ma. This period saw the formation of northward shortening zones, which included upright folds (670 to 610 Ma) and northwest-oriented strike-slip shear zones (640 to 560 Ma)^20^. Notable examples of these geological structures include the Najd fault system, the Hamisana Shear Zone, and the Oko shear zone^15,20,140^.

The geometry and kinematic characteristics of the extensive N-S shear zone in the Egyptian Nubian Shield draw attention to the N-S trending Keraf suture in the evolution of the Nubian Shield. This suture, referred to as the Keraf suture, marks the boundary between the Arabian-Nubian Shield (ANS) and the Saharan Metacraton in northeastern Kenya and eastern Sudan (Fig. 22d **and e**), effectively separating the low-grade Neoproterozoic rocks of the Arabian Nubian Shield from the high-grade rocks of the Saharan Metacraton^20^^146^,. Keraf ophiolites possess many lithological traits, like those found in Phanerozoic ophiolites, and are positioned as fault-bounded ridges that align in an N-S direction. The Saharan Metacraton, located on the African continent, is characterized by continental fragments and arc terranes accreted along the Keraf suture zone during the interval of 650-600 Ma^148^. This region features an N-S-trending structural belt associated with ophiolites, which emerged from the collision between the Arabian Nubian Shield to the east and the Saharan Metacraton to the west^20,148,149^. In the southern Keraf region (Fig. 22d **and e)**, the landscape is dominated by N-trending upright folds deformed by strike-slip faults oriented from N to NW and NNW^20,149^. The multiphase transpressive deformation that affected the Keraf Zone spanned from approximately 650 to 570 Ma^149^. This process was instigated by the oblique convergence between the Arabian-Nubian Shield (ANS) and the Saharan Metacraton in a NW–SE direction, resulting in the formation of N-trending upright folds in the northern Keraf Zone and N- and NNW-trending sinistral strike-slip faults in the southern Keraf Zone^147^. Sinistral shearing along the KS resulted from oblique convergence, which led to its superimposition by the KSZ around 640-580 Ma^147,149^. Lateral displacement during the late-to-post-collisional period is predominantly observed in the southern section of the KSZ, occurring between approximately 630 and 590 Ma^147,150^. The Keraf shear zone developed concurrently (∼650–570 Ma) with the Najd Fault System and the N-S shear zones in the ENS, suggesting a relationship between this suture and the significant N-S shear zones in the ENS.

## Conclusions

- The Egyptian Nubian Shield is the northeastern extension of the East African Orogeny (EAO), extending from northern Sudan to northern Egypt and covering approximately 100,000 km². Its tectonic history spans over 600 million years. The shield is divided into three major phases: the formation of arc terrains, the subsequent accretion of this arc to the Nile Craton, and the reworking of the arc after accretion.
- The Egyptian Nubian Shield is divided into three distinct regions based on their structural features: the Southern Compressional Domain, the Central Transpressional Domain, and the Northern Extensional Domain. The Southern Compressional Domain is shaped by arc-arc sutures and shear zones associated with syn-accretionary processes. In contrast, the Central Transpressional Domain features deformation zones that form after accretion and are connected to the Najd Fault System (NFS). The Northern Extensional Domain, marked by the Barud shear belt, is characterized by extensional geological structures.
- The shear zones linked to the Najd Fault System (NFS) form a complex network of sinistral strike-slip ductile shear zones oriented NW-SE, mainly found within the central transpressional zone of the ENS. Additionally, the dextral shear zones trending NE-SW to ENE-WSW, such as the Mubarak-Barramiya and Barud shear belts, cross the northwest-oriented fabric, forming a conjugate shear system.
- The large-scale N-S trending shear zones in the Egyptian Nubian Shield include: (i) Safaga-Shalul shear zone (SSSZ), (ii) Wadi Kareim-Umm Bisilla shear zone (KBSZ), (iii) Um Gheig-Nugrus shear zone (GNSZ), (iv) Barramiya-Mueilha shear zone (BMSZ), (v)Abu Swayel- Muqsim shear zone (SMSZ), and (vi) Himitrah-Madari shear zone (HMSZ).
- The N-S shear zones comprise a complex network of ductile shear zones characterized by the presence of mylonite and porphyroclastic mylonite. Within these N-S-oriented ductile shear zones, asymmetrical features in the mylonitic rocks indicate non-coaxial strain. Kinematic indicators indicate a dextral shear sense in the N-S-trending shear zones within the Egyptian Nubian Shield. Conversely, brittle N-S-strike-slip faults exhibit sinistral displacement.
- The structural evolution of N-S shear zones can be divided into five main deformational stages. The first stage involves N-S shortening, characterized by southward thrust imbrications and the development of east-west mineral foliations and folds. This phase marks the initial collision between the South Eastern Desert terrane to the north and the Haya and Gabgaba terranes to the south, which occurred roughly between 830 and 720 Ma. The next phase, marked by NE-SW-directed shortening and NW-trending sinistral shearing, creates kilometer-scale northwest and north-northwest sinistral faults. Subsequently, E-W-directed shortening, combined with N-S dextral shearing, produces N-S-striking fabric and significant folds. Lastly, the E-W-directed shortening and NE-directed dextral shearing generate ENE-WSW and NE-SW dextral shear zones, which play a key role in forming a prominent NW-strike-slip sinistral shear system.
- The central transpressional zone within the ENS is characterized by the Najd Fault System (NFS), a complicated network of sinistral strike-slip ductile shear zones aligned NW-SE, developed between 540 and 620 Ma. During the D3 phase of the ENS, the initial east-west and northwest folds experienced deformation, transforming into north-trending folds due to dextral shearing along N-S shear zones. This period saw the emergence of multiple N-S-trending shear zones, roughly 600-590 Ma, affecting all rock units except post-tectonic granite. The Red Sea rifting reactivated these ductile N-S dextral shear zones, turning them into brittle sinistral strike-slip faults that affected all rock units and structures. These faults are oriented N-S, NW-SE, NE-SW, and E-W. The NE-SW and east-west faults intersect with dextral N-S faults, offsetting the left-lateral NW-SE strike-slip faults.
- The ENS contains N-S-oriented dextral shear zones characterized by a complex network of ductile shear zones that include ophiolitic materials, mylonitic schist belts, and antiforms. These structures stretch for hundreds of kilometers and can be up to 10 km wide. They are associated with transpressional belts within the Egyptian Nubian Shield and significantly influence the east-west (D1) and northwest-southeast (D2) structural fabrics, which are further modified by a NE-SW (D4) shear fabric. The NW-SW, N-S, and NE-SW shear zones in the ENS are conjugate systems that formed during a progressive deformation event associated with the Najd Fault System (NFS). Geophysical evidence indicates these primary shear zones extend beneath the surface and may connect to the extensive N-S striking shear zones in the Nubian Shield, such as the Hamazana shear zone and the Keraf suture. The Keraf shear zone formed around the same time as the Najd Fault System (approximately 650–570 Ma) and the N-S shear zones in the ENS (around 620 to 540 Ma), suggesting a relationship between this suture and the development of the N-S dextral shear zones in the ENS.

## Data Availability

The datasets used and/or analysed during the current study available from the corresponding author on reasonable request.
